# A Facile Direct Route to *N*‐(Un)substituted Lactams by Cycloamination of Oxocarboxylic Acids without External Hydrogen

**DOI:** 10.1002/cssc.201901780

**Published:** 2019-07-17

**Authors:** Hu Li, Hongguo Wu, Heng Zhang, Yaqiong Su, Song Yang, Emiel J. M. Hensen

**Affiliations:** ^1^ State Key Laboratory Breeding Base of Green Pesticide & Agricultural Bioengineering Key Laboratory of Green Pesticide & Agricultural Bioengineering, Ministry of Education, State-Local Joint Laboratory for Comprehensive, Utilization of Biomass Center for R&D of Fine Chemicals Guizhou University Guiyang Guizhou 550025 P.R. China; ^2^ Laboratory of Inorganic Materials & Catalysis, Schuit Institute of Catalysis Department of Chemical Engineering and Chemistry Eindhoven University of Technology P.O. Box 513, 5600 MB Eindhoven The Netherlands

**Keywords:** amination, cyclization, density functional calculations, reduction, lactams

## Abstract

Lactams are privileged in bioactive natural products and pharmaceutical agents and widely featured in functional materials. This study presents a novel versatile approach to the direct synthesis of lactams from oxocarboxylic acids without catalyst or external hydrogen. The method involves the in situ release of formic acid from formamides induced by water to facilitate efficient cycloamination. Water also suppresses the formation of byproducts. This unconventional pathway is elucidated by a combination of model experiments and density functional theory calculations, whereby cyclic imines (5‐methyl‐3,4‐dihydro‐2‐pyrrolone and its tautomeric structures) are found to be favorable intermediates toward lactam formation, in contrast to the conventional approach encompassing cascade reductive amination and cyclization. This sustainable and simple protocol is broadly applicable for the efficient production of various *N*‐unsubstituted and *N*‐substituted lactams.

## Introduction

Lactams are cyclic amides, which form a class of core structural motifs privileged in a variety of bioactive natural products and pharmaceutical agents,^1^ and feature widely in functional materials and catalysts.^2^ Pyrrolidones are a ubiquitous subgroup of lactams, which are of great and longstanding interest in the preparation of five‐membered nitrogen‐containing heterocycles.2a, 3 The regioselective establishment of C−C and C−N bonds is a powerful strategy to build N‐heterocyclic compounds. An attractive approach to secondary lactams is catalytic cyclization of primary amides with intramolecular alkenes in the presence of metal catalysts via aza‐Heck or aza‐Wacker mechanisms, in spite of the need to use, respectively, highly electrophilic or nucleophilic nitrogen species.^4^ Lactams can also be obtained in other ways, such as by combining homoenolates with acid‐activated imines,^5^ intramolecular hydrocarbamoylation of allylic formamides,^6^ Michael addition–proton transfer–enol lactonization of acyl chlorides with dicarbonyls,^7^ intramolecular alkenylation of acyclic bromoalkenes,^8^ and cycloamination of prenyl carbamates and ureas.^9^ All of these approaches require specific catalysts and/or additives, as well as organic solvents, to afford satisfactory yields. Therefore, it would be desirable to develop a catalyst‐ and solvent‐free method for the construction of valuable lactam skeletons from sustainable and low‐cost substrates.

**Scheme 1:**
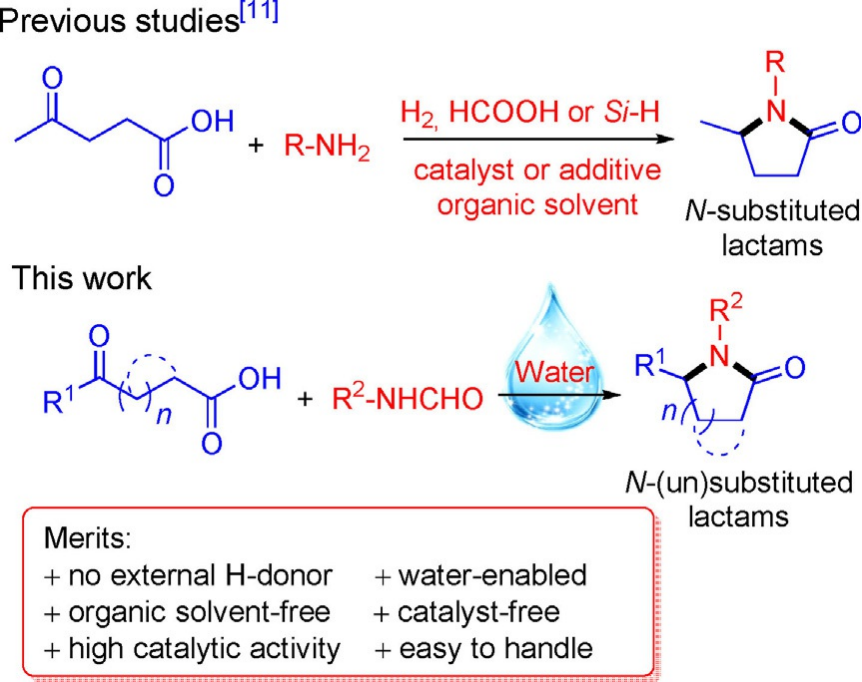
Cycloamination strategies for the synthesis of *N*‐(un)substituted lactams.

Oxocarboxylic acids contain one or more aldehydic or keto groups in a carboxylic acid and are widely available compounds. A prime example is levulinic acid (LA), which can be easily obtained from renewable lignocellulosic biomass.^10^ The keto‐acid LA can be converted with primary amines into *N*‐substituted lactams or pyrrolidones by cascade reductive amination and cyclization catalyzed by transition metals (Scheme 1).^11^ In connection with this, various strategies have recently been proposed to design and prepare catalysts with unique functionalities for simplifying the overall process, such as the use of low‐pressure hydrogen, or hydrogen‐donating co‐reactants such as formic acid (HCOOH) and hydrosilanes under mild conditions.^11^, ^12^, ^13^ Significant progress has been made in the selective production of *N*‐substituted lactams and pyrrolidones from LA (see the Supporting Information, Table S1), but in all cases the use of an organic solvent, an external hydrogen source, and/or additives are key to obtain good results.

Leuckart reductive amination is a well‐known reaction for catalyst‐free upgrading of aldehydes and ketones to formamides by using HCOOH as a hydrogen donor, although it is hampered by low selectivity and high required reaction temperatures (ca. 180 °C).^14^ Efforts to improve the product selectivity and reaction rate include the use of basic additives (e.g., NEt_3_) or microwave heating to activate HCOOH and minimize the negative effect of its acidity during reductive amination.^15^ Herein, we propose a novel and generic strategy involving in situ‐controlled release of HCOOH from *N*‐formyl species with deionized water for cyclic diamination of LA and other keto‐acids. Our synthesis protocol provides access to various *N*‐(un)substituted lactams without catalyst or external hydrogen and requires no organic solvent (Scheme 1).

## Results and Discussion

We explored the use of formamide (H_2_NCHO) and HCOOH as nitrogen and hydrogen sources, respectively, for the cycloamination of LA (without catalyst or solvent) and obtained a moderate 5‐methyl‐2‐pyrrolidinone (MPD) yield of 83 % after reaction for 1.5 h at 160 °C (Table 1, entry 1). From a green chemistry point of view, the tenfold excess of HCOOH results in undesired waste with respect to process mass intensity (PMI=8.7; Table 1, entry 1) and E‐factor (5.0; Table S2) parameters. Without HCOOH, the MPD yield decreased to 41 % at 160 °C (Table 1, entry 2), implying the possibility of in situ release of HCOOH from H_2_NCHO or other *N*‐formyl intermediates during the reaction. To explore this opportunity, several control experiments were conducted. First, LA can be directly cyclized to form angelica lactones (ALs) by dehydration.^16^ Second, H_2_NCHO can be hydrolyzed in water with conversions up to around 70 % after 1.5 h at 160 °C and were then constant upon prolonged reaction to 4 h. These findings demonstrate that water obtained by LA dehydration can in principle promote the hydrolysis of H_2_NCHO and relevant *N*‐formyl intermediates to release HCOOH. As expected, the addition of water (30 equiv) into the reaction systems with and without HCOOH (Table 1, entries 3 and 4) significantly accelerates the cycloamination reaction, providing comparable MPD yields of 92 and 94 %. It is worth noting that the reaction without HCOOH exhibits superior green chemistry metrics, including a high reaction mass efficiency (RME), a low E‐factor, and good atom economy compared to the case with HCOOH (Table S2). The PMI of the present protocol that avoids the use of HCOOH is 8.5 (Table 1, entry 4), which is much lower than the values between 20 and 199 for other reported approaches (Table 1, entries 5–11).^11^, 12d, 13c, 17 These features render our synthetic method highly promising for the sustainable production of lactams in good yield.

**Table 1:** Catalytic cycloamination of LA to lactams using different systems.

Entry	Catalyst	Nitrogen source	Hydrogen source	Additive/Solvent	*T* [°C]	*t* [h]	Lactam	Yield [%]	PMI^[c]^	Rate^[d]^	Ref.
1^[a]^	–	H_2_NHCO	HCOOH	–	160	1.5	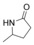	83	8.7	1107	this work
2^[a]^	–	H_2_NHCO	–	–	160	4		41	6.2	205	
3^[b]^	–	H_2_NHCO	HCOOH	H_2_O	160	1.5		92	13.7	1227	
4^[b]^	–	H_2_NHCO	–	H_2_O	160	4		94	8.5	470	
5	–	benzyl amine	HCOOH	NEt_3_/ DMSO	100	12	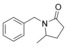	87	199	73	13c
6	Pt‐MoO_*x*_/TiO_2_	*n*‐octyl amine	3 bar H_2_	–	100	20	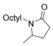	99	66	50	12d
7	Ru‐P complex	benzyl amine	HCOOH	–	120	12	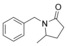	95	90	79	17
8	In(OAc)_3_	R‐NH_2_	PhSiH_3_	toluene	120	1‐24	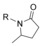	49‐97	>20	180	11b
9	Pt/P‐TiO_2_	aqueous NH_3_	1 bar H_2_	MeOH	25	72	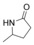	85	32	12	11a
10	Pt/P‐TiO_2_	5 bar NH_3_ gas	15 bar H_2_	MeOH	25	72		87	38	12	11a
11	Au/ZrO_2_‐VS	aqueous NH_3_	HCOOH	H_2_O	130	16		85	45	53	11c

[a] LA/H_2_NHCO/HCOOH/H_2_O molar ratio=1:3:10:30. [b] LA/H_2_NHCO/H_2_O molar ratio=1:6:30. [c] Process mass intensity (PMI) is defined as (mass of raw materials input)/(mass of desired output). [d] Reaction rate defined as moles of product/time (μmol h^−1^)

Table 1 shows that systems depending on metal catalysts require much longer reaction times (up to 72 h) to achieve moderate yields of either substituted or unsubstituted lactams (entries 8–11), despite relatively low reaction temperatures (25–130 °C).^11^ Our method shows superior performance in terms of the reaction rate with and without HCOOH (1227 and 470 μmol h^−1^; Table 1, entries 3 and 4) while affording good lactam yields that are comparable to previously reported results (entries 3–11). The significantly higher MPD formation rate with HCOOH indicates that hydrogen transfer may be the rate‐determining step. Therefore, we hypothesized that optimizing the reaction conditions towards faster in situ release of HCOOH from H_2_NCHO and the involved *N*‐formyl species assisted by water can improve the reaction performance without HCOOH. The use of 6 equivalents of H_2_NCHO and 30 equivalents of water resulted in a satisfactory MPD yield of around 90 % from LA at 160 °C after 2 h (Figures S1 and S2). The reaction temperature and time strongly impact the MPD formation rate and product distribution (Figure S3). Specifically, unreduced 5‐methyl‐3,4‐dihydro‐2‐pyrrolone (MDPY) and nitrogen‐free γ‐valerolactone (GVL) were observed as byproducts in the novel reaction system (Figures S4 and S5), alongside previously reported 4‐formamidopentanoic acid (FPAC) and 4‐formamidopentanamide (FPAM) intermediates.11b, 12d, 18 These findings suggest that the reducing and C−N coupling functionalities of the reaction system are responsible for the product distribution, which lies between typical catalyst‐mediated reductive amination and our novel approach free from catalyst and external hydrogen.

**Figure 1:**
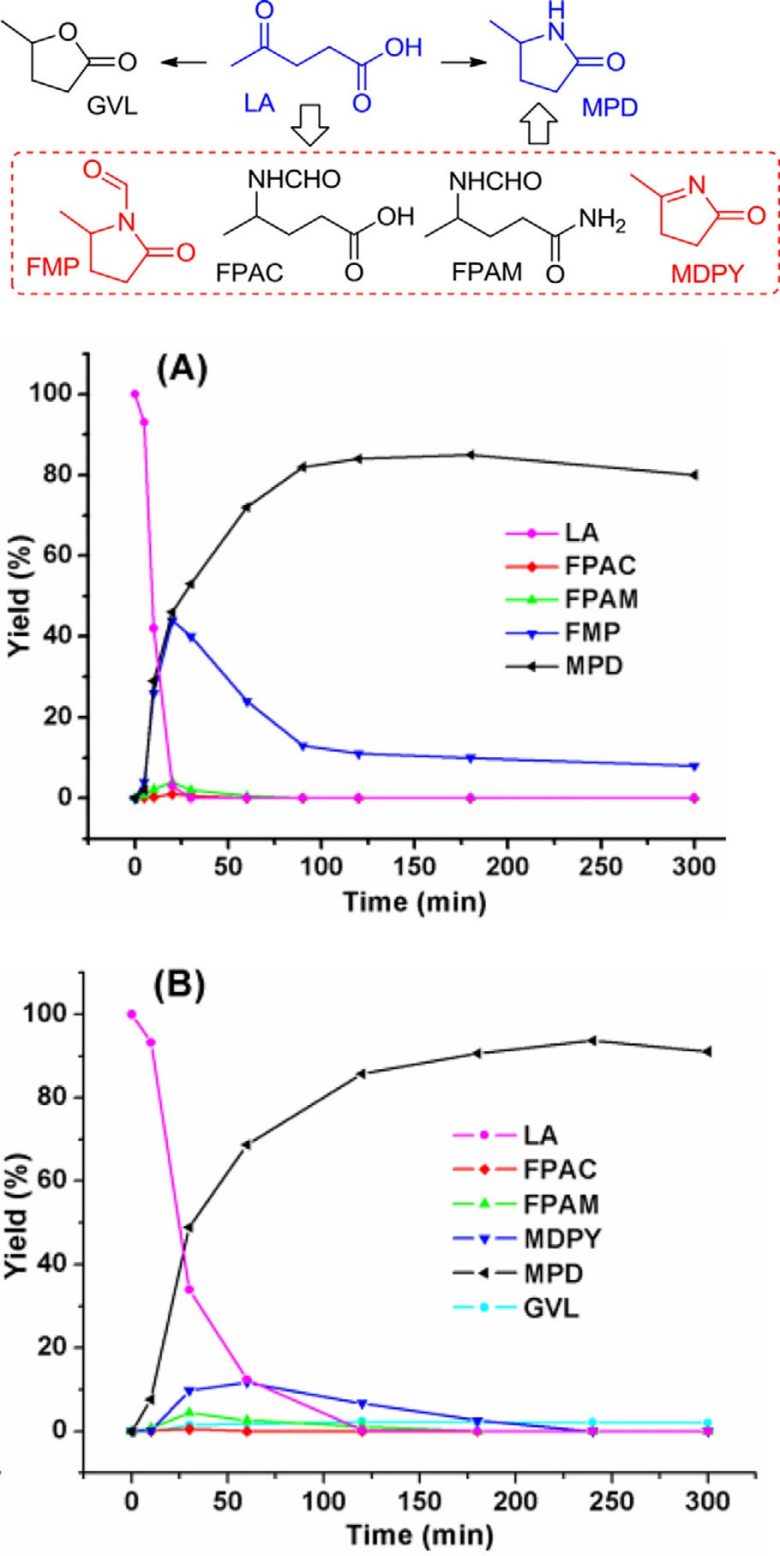
Product distribution in cycloamination of LA with H_2_NCHO assisted by HCOOH (A) or water (B) heated at 160 °C for variable times.

As follows from Table 1, the thermal reaction system of LA and H_2_NCHO with HCOOH (1107 μmol h^−1^) instead of water (470 μmol h^−1^) results in a superior MPD formation rate (entry 1 vs. 4), highlighting the significance of hydrogen transfer in the overall cycloamination reaction. Although both types of reactions proceed via the key intermediates FPAC and FPAM, the reaction with excess HCOOH affords another cyclic compound *N*‐formyl 5‐methyl‐2‐pyrrolidinone (FMP) as a main byproduct (Figure 1). We also observe that the presence of HCOOH substantially decreases the time to completely convert LA (20 min), in comparison with the water‐assisted system (120 min). Notably, the formation of the relatively stable FMP results in a low MPD yield (Figure 1). Water suppresses the formation of FMP by hydrolysis, thus improving the MPD yield (Table 1, entry 1 vs. 3). Thus, we can infer that water is not only favorable for in situ release of HCOOH by hydrolysis of H_2_NCHO and *N*‐formyl intermediates, but also avoids the formation of undesired species like FMP that are chemically stable under anhydrous conditions.

The formation of GVL (Figures 1 B and S3), which can be obtained from ALs by hydrogenation with HCOOH,^19^ suggests that ALs could be the intermediates leading to the N‐heterocycles MDPY and MPD. To explore this possibility, α‐AL was thermally treated with H_2_NCHO in water or neat conditions, whereby 44 % and <6 % MPD yields together with MDPY in roughly 8 % and <1 % yields, respectively, were obtained after heating at 160 °C for 1 h. These differences can be attributed to the reversibility of the reaction between LA and ALs,^16^ with the presence of water shifting the equilibrium to the LA side. LA is the species that reacts to form MPD and MDPY. A kinetic study of the conversion of LA into MPD with normal and deuterated water reveals a secondary hydrogen isotope effect with *k*_H_/*k*_D_=0.94 (Figure S6). The equilibrium deuterium isotope effect suggests reversible processes in the overall conversion of LA into MPD,^20^ especially those involved with water.

Considering the possible activating role of the acidic carboxyl group (‐COOH) of LA in the synthesis of MPD by cycloamination, ethyl levulinate (EL) was used as a substrate instead of LA, affording MPD in around 90 % yield (Figure S7). A kinetic study showed comparable reaction rate constant (*k*_LA_/*k*_EL_=1.05) for cycloamination of LA (0.0519 min^−1^) and EL (0.0493 min^−1^; Figure S8), demonstrating that the acidity of the substrate has a minor effect on the reaction outcome. We also investigated the order of amination and amidation steps in the conversion of LA into MPD by examining the reactivity of 2‐pentanone and pentanoic acid under otherwise similar conditions (Figure S9). The results clearly show that amination is much faster than amidation independent of the presence of HCOOH. Another salient detail of this experiment was that the conversions of 2‐pentanone and pentanoic acid were both lowered in water in comparison with the HCOOH‐mediated reaction (Figure S9), further revealing that the rate‐determining step in the cycloamination without external hydrogen source is likely the in situ release of HCOOH from H_2_NCHO and involved *N*‐formyl species assisted by water.

**Figure 2:**
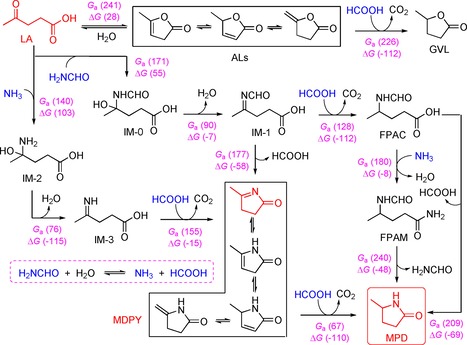
Cycloamination of LA with H_2_NCHO and water: (A) Reaction pathways and (B) computed free energy profiles. IM: intermediate; TS: transition state. Values in parentheses are free energies and enthalpies (kJ mol^−1^).

The hydrogen transfer process and the presence of ALs and MDPY tautomeric structures were investigated by deuterium labeling experiments (Figures S10–S12). The large body of reaction data allowed us to infer the reaction pathways for LA cycloamination with H_2_NCHO and water (Figure 2 A). Initially, H_2_NCHO is reversibly hydrolyzed to NH_3_ and HCOOH under hydrothermal conditions with a reported free energy of activation of around 146 kJ mol^−1^,^21^ whereas LA is able to undergo either C−N coupling with H_2_NCHO to afford 4‐(formylimino)pentanoic acid (IM‐1) via IM‐0 or intramolecular dehydration to give ALs with tautomeric structures, respectively, owing to insufficient HCOOH derived from H_2_NCHO hydrolysis in the early stage. With the assistance of the in situ*‐*formed HCOOH, a limited amount of ALs is hydrogenated to GVL (Figure 2 A). As the dominant reaction pathway, IM‐1 is more prone to release HCOOH and undergo intramolecular cyclization, giving a favorable precursor MDPY with relevant tautomeric structures that are gradually transformed into MPD by hydrogen transfer during prolonged reaction. In parallel, IM‐1 may undergo cascade transfer hydrogenation (FPAC), amidation (FPAM), and cyclization to afford the MPD product, despite the low rates of these reactions.

To examine whether conversion of LA into MPD proceeding through MDPY is kinetically and thermodynamically favorable, density functional theory (DFT) calculations were performed at the B3LYP/6‐311+G(2s,2p) level in conjunction with polarizable continuum model (PCM) to simulate the solvent effect (Figures 2 and S13). LA can react with H_2_NCHO to obtain intermediate IM‐0 by overcoming an activation free energy barrier (*G*_a_) of 171 kJ mol^−1^, followed by conversion to IM‐1 through releasing one water molecule (*G*_a_=90 kJ mol^−1^). From IM‐1 to the product MPD via the precursor MDPY involving the release of HCOOH, the highest activation energy barrier is calculated to be 177 kJ mol^−1^, in good agreement with the experimental activation energy of 162 kJ mol^−1^ (Figure S14). In contrast, the pathways via the precursor FPAC and FPAM toward MPD require 32 kJ mol^−1^ and 63 kJ mol^−1^ higher activation energy, respectively, clearly demonstrating that the pathway involving MDPY is preferred. Notably, FPAM formation requires a comparable activation energy of 180 kJ mol^−1^, indicative of the possibility of affording FPAM from IM‐1 via FPAC, as identified by GC‐MS (Figure S4), which is also consistent with the experimental results (Figures 1 and S3). In view of gaseous NH_3_ and HCOOH being simultaneously formed in a certain amount by thermal hydrolysis of H_2_NCHO, the initial amination of LA with NH_3_ instead of H_2_NCHO was also taken into consideration (Figure 2). The energy barrier for the reaction between LA and NH_3_ to afford MDPY successively via IM‐2 and IM‐3 was calculated to be 155 kJ mol^−1^, which is slightly lower than the latter (177 kJ mol^−1^). It is worth noting that the used reactants LA (b.p.≈245 °C) and H_2_NCHO (b.p.≈210 °C) are both in the liquid phase during the reaction process at a temperature of 160 °C. Therefore, we may expect that amination occurs between these components as well as between liquid LA and NH_3_ (gas), considering mass transfer limitations of gas–liquid‐phase reactions.^22^ In addition, AL formation hinders LA dehydration (Figure 2 and S13), as proven by the poor GVL yield. These results elaborate that cyclic imines (MDPY with its tautomeric structures) derived from LA by cycloamination are preferentially converted into the lactam by subsequent hydrogen transfer, which is totally different to the typical cascade reductive amination and cyclization process.

Encouraged by the prominent performance of the developed reaction system, the substrate scope with respect to functional group tolerance was further examined (Table 2). Apart from H_2_NCHO (Table 2, entry 1), ammonium formate (HCOONH_4_) was found to exhibit much higher reactivity (entry 2), leading to shorter reaction time (3 h) and higher MPD yield (97 %). In comparison with H_2_NCHO, the increasing hydrolysis ability of ammonium formate can contribute to the superior performance, also proving that the release of HCOOH from thermal treatment of H_2_NCHO and involved *N*‐formyl species is the rate‐determining step. However, LA promoted by a 1:1 mixture of aqueous HCOOH and NH_3_ only gave MPD in a low yield of around 70 % under identical conditions, whereas increased amounts of GVL and formylated/condensed coproducts were formed. Even worse, when primary amines (e.g., propylamine, cyclohexylamine, aniline, 3‐methylaniline, 4‐methylaniline, and benzylamine) and HCOOH were used as substrates for the cascade aqueous reaction processes, only 10–50 % yields of *N*‐substituted lactams were obtained under optimized conditions. These results indicate that the in situ release of HCOOH from H_2_NCHO, HCOONH_4_, or other *N*‐formyl species assisted by water is more favorable for suppressing the occurrence of side reactions to ensure relatively high selectivity toward MPD. When the 4‐substituent of the oxocarboxylic acid substrate is changed from methyl to phenyl, 4‐chlorophenyl, 4‐fluorophenyl, and 2‐thienyl groups, the corresponding unsubstituted lactams are obtained in good yields (81–93 %; Table 2, entries 3–6). Alongside the 1,4‐dicarbonyl substrates, a range of 1,5‐ and 1,6‐dicarbonyl compounds can be also subjected to cycloamination, affording moderate to good yields (68–92 %) of six‐ and seven‐membered lactams, respectively (Table 2, entries 7–10). Importantly, unsubstituted benzolactams (62–80 %) can be obtained from *o*‐phenyl dicarbonyl compounds with H_2_NCHO assisted by water (Table 2, entries 11 and 12). In addition to H_2_NCHO, a series of other formamides that can be simply synthesized by catalyst‐free *N*‐formylation of amines with CO_2_ 23 were also employed as both nitrogen and hydrogen sources, and relevant *N*‐substituted lactams can be obtained with satisfactory yields (75–98 %; Table 2, entries 13–22).

**Table 2:** Production of *N*‐(un)substituted lactams from various oxocarboxylic acids and formamides.^[a]^ 
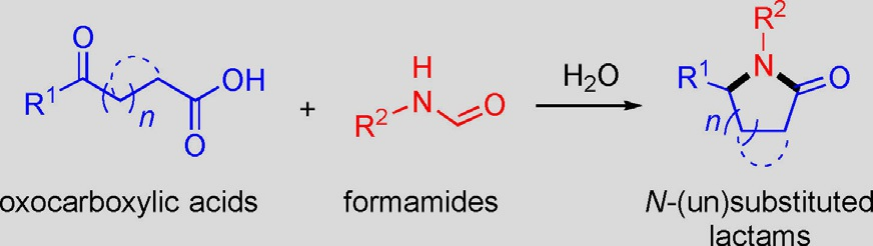

Entry	Oxocarboxylic acid		Formamide	Lactams	*t* [h]	Yield [%]
	R^1^	*N*	R^2^			
1	CH_3_	1	H	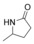	4	94
2^[b]^	CH_3_	1	HCOONH_4_		3	97
3	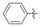	1	H	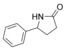	4	81
4	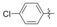	1	H	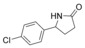	5	86
5	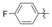	1	H	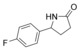	6	87
6	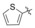	1	H	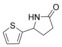	4	93
7	CH_3_	2	H	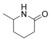	5	92
8	CH_3_	3	H	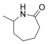	5	68
9	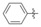	2	H	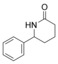	4	85
10	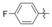	2	H	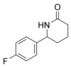	4	70
11	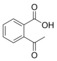		H	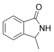	5	80
12	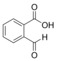		H	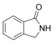	5	62
13	CH_3_	1	CH_3_	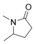	4	98
14	CH_3_	1	CH_3_CH_2_	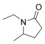	4	94
15	CH_3_	1	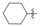	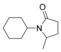	4	80
16	CH_3_	1	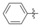	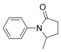	4	85
17	CH_3_	1	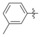	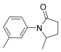	4	83
18	CH_3_	1	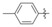	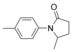	4	84
19	CH_3_	1	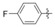	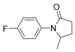	5	75
20	CH_3_	1	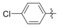	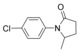	5	78
21	CH_3_	1	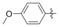	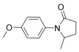	4	89
22	CH_3_	1	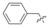	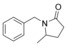	4	88

[a] Reaction conditions: Oxocarboxylic acid (2 mmol), formamide (6 equiv), water (30 equiv), 160 °C. [b] HCOONH_4_ instead of H_2_NCHO was used as the nitrogen source.

To evaluate the potential practical implementation of our new method, the efficiency of the catalyst‐ and external hydrogen‐free system in the cycloamination of LA with H_2_NCHO and water was examined in a continuous‐flow microreactor. Under the optimized reaction conditions (LA/H_2_NCHO/H_2_O molar ratio=1:6:30, *T*=160 °C), a good MPD yield of 87 % was obtained with a residence time of 20 min. In comparison with the established batch procedure (Table 1, entry 4), the flow reactor shortens the reaction duration, with superior reaction rate and comparable lactam yield.

## Conclusions

In summary, a facile versatile approach to directly synthesize both *N*‐unsubstituted and *N*‐substituted lactams under catalyst‐ and external hydrogen‐free conditions has been developed involving in situ release of HCOOH from formamides promoted by water for efficient cycloamination. Experimental and computational results show that cyclic imines are the key species toward the lactam by undergoing subsequent hydrogen transfer. This route is completely different to typical catalytic reaction pathways, involving reductive amination followed by cyclization. The novel benign and versatile protocol exhibits good universality and applicability in lactam synthesis. We envision it to find broader application by giving access to various nitrogen‐containing compounds, especially high‐value N‐heterocycles.

## Experimental Section

### Reaction procedures

All experiments were carried out in a Teflon‐lined stainless steel autoclave (inner volume 15 mL), placed in an oil bath that was preheated to the desired reaction temperature (120–180 °C). In a typical reaction procedure, LA or keto‐acid (2 mmol), H_2_NCHO (12 mmol), or formamides (6 equiv), and deionized water (60 mmol, 30 equiv) were added into the autoclave and the reaction duration was recorded as the autoclave was placed into the oil bath. After a specific reaction time, the autoclave was taken out of the oil bath and immediately cooled‐down to ambient temperature with tap‐water. Upon completion, the autoclave was opened, and deionized water (or methanol) was added to the reaction mixture to dilute it to 25 mL before it was subsequently analyzed by HPLC (or GC). Each experiment was separately conducted and repeated 2 or 3 times. The obtained conversions and yields are average data of 2 or 3 individual experiments, with standard deviation (*σ*) in the range of 0.5–4.6 %. Structures were confirmed by ^1^H and ^13^C NMR spectroscopy (JEOL‐ECX 500 NMR spectrometer, CDCl_3_), GC‐MS, and HRMS.

For product separation from the reaction mixture, CH_2_Cl_2_ or ethyl acetate can be used as an effective extractant. Typically, deionized water (3–5 mL) was added into the mixture after the reaction and the product lactam could be isolated by extraction with CH_2_Cl_2_ or ethyl acetate (3×5 mL). Solvent was removed from the resulting combined extract by evaporation under reduced pressure to give the product lactam.

For continuous‐flow reactions, a Labtrix Start microreactor system (Chemtrix BV, NL) with a glass micro reactor (type 3227, volume: 19.5 μL) was utilized. Initially, LA, H_2_NCHO, and deionized water in a molar ratio of 1:6:30 were evenly mixed and added into a flask. The resulting solution was pumped into the microreactor (rate: 25 μL min^−1^) under cooling until the reactor was full, whereupon the reactor temperature was raised to 160 °C and the solution flow rate was set at 1.5 μL min^−1^. After running for 1.5 h, sampling at timed intervals was conducted for GC analysis.

### Computational methods

All DFT calculations were carried out using the hybrid functional B3LYP^24^ as implemented in Gaussian 09 D.01 software.^25^ The all‐electron 6‐311+G(d,p) basis set was used for all atoms. The polarized continuum model (PCM)^26^ with standard parameters for water solvent (*ϵ*=78.3) was used to account for bulk solvent effects during geometry optimization and searching of transition states. Frequency analysis was performed to ensure that each transition state has only one imaginary frequency in the direction of the reaction coordinate. All relative energies discussed herein are referred to Gibbs free energies considered the zero‐point energy (ZPE) correction at 453 K.

## Conflict of interest

*The authors declare no conflict of interest*.

## Supporting information

As a service to our authors and readers, this journal provides supporting information supplied by the authors. Such materials are peer reviewed and may be re‐organized for online delivery, but are not copy‐edited or typeset. Technical support issues arising from supporting information (other than missing files) should be addressed to the authors.

Supplementary
