# Supplementary material for: A Facile Direct Route to N‐(Un)substituted Lactams by Cycloamination of Oxocarboxylic Acids without External Hydrogen
Source: ChemSusChem. 2019 Jul 17;12(16):3778–84. doi: 10.1002/cssc.201901780 (PMC6772168; doi:10.1002/cssc.201901780)
Supplement: Supplementary file 1 — Supplementary [file CSSC-12-3778-s001.pdf]

CHEMISTRY & SUSTAINABILITY
CHEMSUSCHEM
ENERGY & MATERIALS

Supporting Information

A Facile Direct Route to *N*-(Un)substituted Lactams by Cycloamination of Oxocarboxylic Acids without External Hydrogen

Hu Li+,[a] Hongguo Wu+,[a] Heng Zhang,[a] Yaqiong Su,*[b] Song Yang,*[a] and Emiel J. M. Hensen*[b]

cssc_201901780_sm_miscellaneous_information.pdf

Experimental

Materials

Ethyl levulinate (EL, ≥98%), 3-benzoylpropionic acid (99%), *N*-methylformamide (99%), deuterium oxide (D_2O, 99.994 atom % D), formamide (H_2NCHO, ≥99.5%), 4-oxo-4-(2-thienyl)butyric acid (97%), 4-acetylbutyric acid (97%), 2-acetylbenzoic acid (99%), 3-(4-fluorobenzoyl)propionic acid (97%), 5-methyl-2-pyrrolidone (MPD, 98%), angelica lactones (ALs, ≥98.5%), 2-pentanone (≥99%), and pentanoic acid (≥99.8%) were purchased from Sigma-Aldrich (Shanghai). Levulinic acid (LA, 99%), 6-oxoheptanoic acid (96%), 4-(4-fluorobenzoyl)butyric acid (98%), *N*-cyclohexylformamide (>98.0%), 4'-methoxyformanilide (>98.0%), chloroform-d_1 (99.96 atom % D), formanilide (99%), 4'-methylformanilide (>98.0%), *N*-formylethylamine (≥97.0%), 3-(4-chlorobenzoyl)propionic acid (>98%), *N*-(4-fluorophenyl)formamide (>98.0%), *N*-(4-chlorophenyl)formamide (>98.0%), and DMSO-d_6 (99.9 atom % D) were bought from Aladdin Industrial Inc. (Shanghai). 2-Carboxybenzaldehyde (>98%), ammonia solution (28% NH_3 in H_2O), 3-methylformanilide (97%), formic acid (HCOOH, 99%), *N*-benzylformamide (98.0%), 4-benzoylbutyric acid (97%), ethyl acetate (99.8%), ammonium formate (99%), CH_2Cl_2 (99.9%), and methanol (99.9%) were supplied from Innochem Inc. (Beijing).

Reaction procedures

All the experiments were carried out in a Teflon-lined stainless steel autoclave (inner volume 15 mL), placed in an oil-bath that was preheated to the desired reaction temperature (120-180 °C). In a typical reaction procedure, 2 mmol LA or keto acid, 12 mmol H_2NCHO or formamides (6 equiv.), and 60 mmol deionized water (30 equiv.) were added into the autoclave, and the reaction duration was recorded as the autoclave was placed into the oil-bath. After a specific reaction time, the autoclave was taken out of the oil bath and immediately cooled-down to ambient temperature with tap-water. Upon completion, the autoclave was opened and deionized water (or methanol) was added to the reacted mixture, which was subsequently analyzed by HPLC (or GC). Each experiment was separately conducted and repeated for 2-3 times. The obtained conversions and yields are average

data of 2-3 individual experiments, with standard deviation (σ) in the range of 0.5-4.6%. Structures were confirmed by ^{1}H, ^{13}C NMR (JEOL-ECX 500 NMR spectrometer, $CDCl_3$), GC-MS, and HRMS.

For the product separation from the reaction mixture, CH_2Cl_2 or ethyl acetate can be used as an effective extractant. Typically, 3-5 mL deionized water was added into the mixture after the reaction, while the product lactam could be isolated by extraction with CH_2Cl_2 or ethyl acetate for 3 times (5 mL × 3). The resulting combined extractant was evaporated under reduced pressure to give the product lactam.

Regarding the continuous-flow reactions, a Labtrix®Start microreactor system (Chemtrix BV, NL) with a glass micro reactor (type 3227, volume: 19.5 μL) was utilized. Initially, LA, H_2NCHO, and deionized water in a molar ratio of 1:6:30 was evenly mixed and added into a flask. The resulting solution was pumped into the micro reactor (rate: 25 μL/min) under cooling until the reactor is full. Upon the reactor temperature was raised to 160 °C, the solution flow rate was set at 1.5 μL/min. After running for 1.5 h, sampling at timed intervals was conducted for GC analysis.

Product analysis

Liquid samples were identified by GC-MS (Agilent 6890N GC/5973 MS, Santa Clara, CA). Concentrations of LA and HCOOH were analyzed by HPLC (Agilent 1260) fitted with a Hi-Plex column and a refractive index (RI) detector. Quantitative analyses of the commercially available and isolated samples were made by GC (Agilent 7890B) with an HP-5 column (30 m × 0.320 mm × 0.25 μm) and a flame ionization detector using naphthalene as internal standard. ^{1}H NMR was used for quantification of unpurified products in the reaction mixtures (diluted with $CDCl_3$ or DMSO-d_6) using 1,3,5-trimethoxybenzene as the internal standard.

For the structural identification of products, HRMS (ESI) analysis was measured on a Q Exactive LC-MS/MS (Thermo Scientific) instrument. ^{1}H and ^{13}C NMR spectra of samples were recorded in DMSO-d_6 or $CDCl_3$ on Bruker NMR spectrometers at 400 MHz and 101 MHz respectively, JOEL ECX-500M spectrometers at 500 MHz and 125 MHz using tetramethylsilane (TMS) as the internal standard. The following abbreviations were used to designate chemical shift multiplicities: s (singlet), d (doublet), t (triplet) and m (multiplet).

In situ ATR-IR analytic experiments

In situ ATR-IR analytic experiments were conducted on a VERTEXV 80V FT-IR spectrometer (Bruker) fitted with a liquid N_2-cooled MCT detector. The equipment is connected to a stainless steel autoclave (100 mL) for the hydrothermal reactions through an optical fiber with a high temperature Diamond ATR probe (Bruker), where an *in situ* mid-infrared based system monitors the reaction in real time. In a general procedure, LA (10 mmol), H_2NCHO (6 equiv.), and deionized water (30 equiv.) were placed into the autoclave and then thermally treated at 160 °C for 4 h. During ATR-IR analysis, the spectra were recorded every 20 min in the wavenumber of 4000-400 cm^{-1}.

Isotopic labeling experiments

GC-MS spectra of the reaction mixtures were used in the experiments under the given reaction conditions with either normal or deuterium water. Analyses were performed after dilution with methanol to determine the incorporated D in the products.

Computational methods

All DFT calculations were carried using the hybrid functional B3LYP [S1a] as implemented in Gaussian 09 D.01 software [S1b]. The all-electron 6-311+G(d,p) basis set was used for all atoms. The polarized continuum model (PCM) [S1c] with standard parameters for water solvent (ε = 78.3) was used to account for bulk solvent effects during geometry optimization and searching of transition states. Frequency analysis was performed to ensure that each transition state has only one imaginary frequency in the direction of the reaction coordinate. All relative energies discussed in this paper are referred to Gibbs free energies considered the zero point energy (ZPE) correction at 453 K.

Table S1. Previous results in LA-to-lactams conversion with different catalysts and H-donors

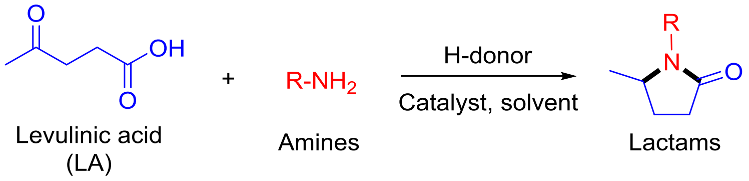

Entry	Catalyst	**R**	Solvent	H-donor	Temp. (°C)	Time	LA conv.(%)	Lactam yield(%)	Ref.
1	Au/ZrO_2-VS	**Benzyl**	H_2O	HCOOH	130	12 h	98	97	S2
2	Ru complex	**Benzyl**	-	HCOOH	120	12 h	-	95	S3
3	Ir complex	**Benzyl**	H_2O	HCOOH/ HCCONa	80	4 h	-	86	S4
4	Pt-MoO_x/TiO_2	**Benzyl**	-	3 bar H_2	100	20 h	-	94	S5
5	Pt/TiO_2	**Phenyl**	-	10 bar H_2	120	18 h	100	100	S6
6	FeNi alloy	**Phenethyl**	EtOH	50 bar H_2	150	WHSV 39 h^{-1}	92	84	S7
7	Ru complex	**Propyl**	-	HCOOH	140	12 h	86	86	S8
8	$Ru_3(CO)_{12}$	**Benzyl**	-	HCOOH	120	12 h	100	93	S9
9	CNF_x@Ni@CNTs	**Benzyl**	GVL	30 bar H_2	130	6 h	100	99	S10
10	[BMIm][Lac]	**Phenyl**	-	$(EtO)_3SiH$	80	1 h	-	91	S11
11	Ir/SiO_2-SO_3H	**Phenyl**	EA	>17 bar H_2	100	24 h	-	88	S12
12	Pt-MoO_x/TiO_2	***n*-Octyl**	-	7 bar H_2	110	24 h	-	92	S13
13	Cp*Ir-L	**Phenyl**	H_2O	25 bar H_2	80	10 h	-	96	S14
14	Ir complex	**Phenyl**	-	5 bar H_2	110	16 h	-	98	S15
15	$AlCl_3·6H_2O$	**Phenyl**	-	$PhSiH_3$	30	12 h	-	93	S16
16	Raney Ni	**Phenyl**	H_2O	HCOOH	180	6 h	100	92	S17
17	$Cu_{15}Pr_3$/Al_2O_3	***n*-Butyl**	DXA	50 bar H_2	175	20 h	99.6	94.2	S18
18	$Fe_3(CO)_{12}$	**Ethyl**	H_2O	HCOOH	180	15 h	-	90	S19
19[a]	-	**Benzyl**	DMSO	HCOOH	100	12 h	-	87	S20
20[b]	-	H	-	-	160	4 h	100	94	This work

GVL: γ-valerolactone; EA: ethyl acetate; DXA: 1,4-dioxane; DMSO: dimethyl sulfoxide.

[a] 1 equiv. Et_3N was added as additive, relative to LA.

[b] 30 equiv. deionized water was added, relative to LA.

Table S2. A quantitative comparison with green chemistry metrics for the synthesis of MPD

Entry	Reactant				Solvent	Additive	Yield	RME	E-factor	Atom economy
	A	B	C	A/B/C[a]						
1	LA	H_2NCHO	HCOOH	1:10:3	-	-	81.9%	0.13	5.0	0.48
2	LA	H_2NCHO	HCOOH	1:10:3	-	H_2O	92.8%	0.11	5.0	0.48
3	LA	H_2NCHO	-	1:6	-	-	41.3%	0.16	2.3	0.61
4	LA	H_2NCHO	-	1:6	-	H_2O	93.7%	0.37	2.3	0.61

[a] Optimized molar ratio of A:B:C

Reaction mass efficiency (RME) = [molecular weight (MW) of product × yield] / [MW of reactant A + (MW of reactant B × molar ratio B/A) + (MW of reactant C × molar ratio C/A)]

E-factor = total waste (g) / product (g)

Atom economy = MW of product × 100% / Σ (MW of reactants)

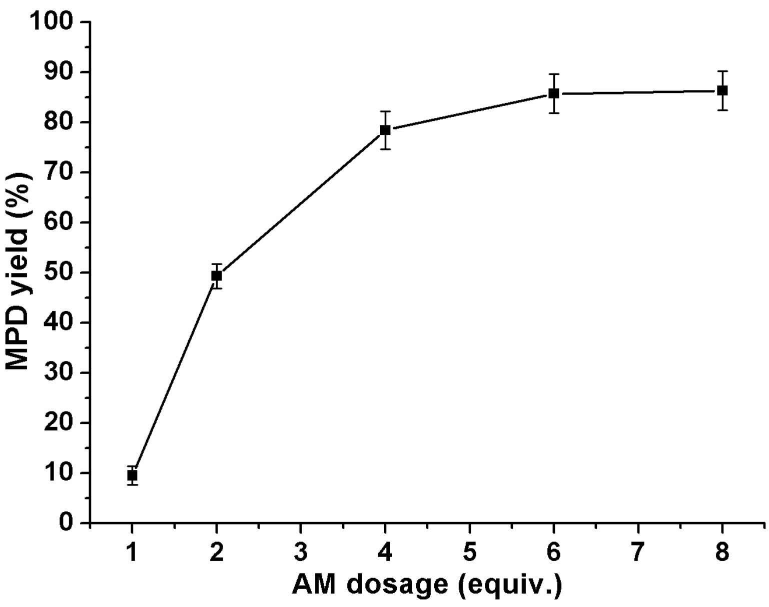

Figure S1. Effect of H_2NCHO dosage on the synthesis of MPD from LA. Conditions: 2 mmol LA, 30 equiv. H_2O, 160 °C, and 2 h.

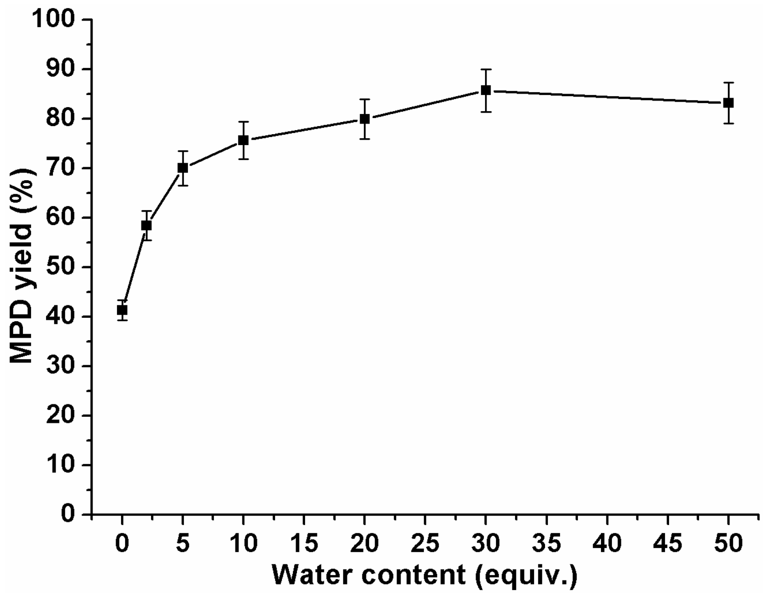

Figure S2. Effect of water dosage on the synthesis of MPD from LA. Conditions: 2 mmol LA, 6 equiv. H_2NCHO, 160 °C, and 2 h.

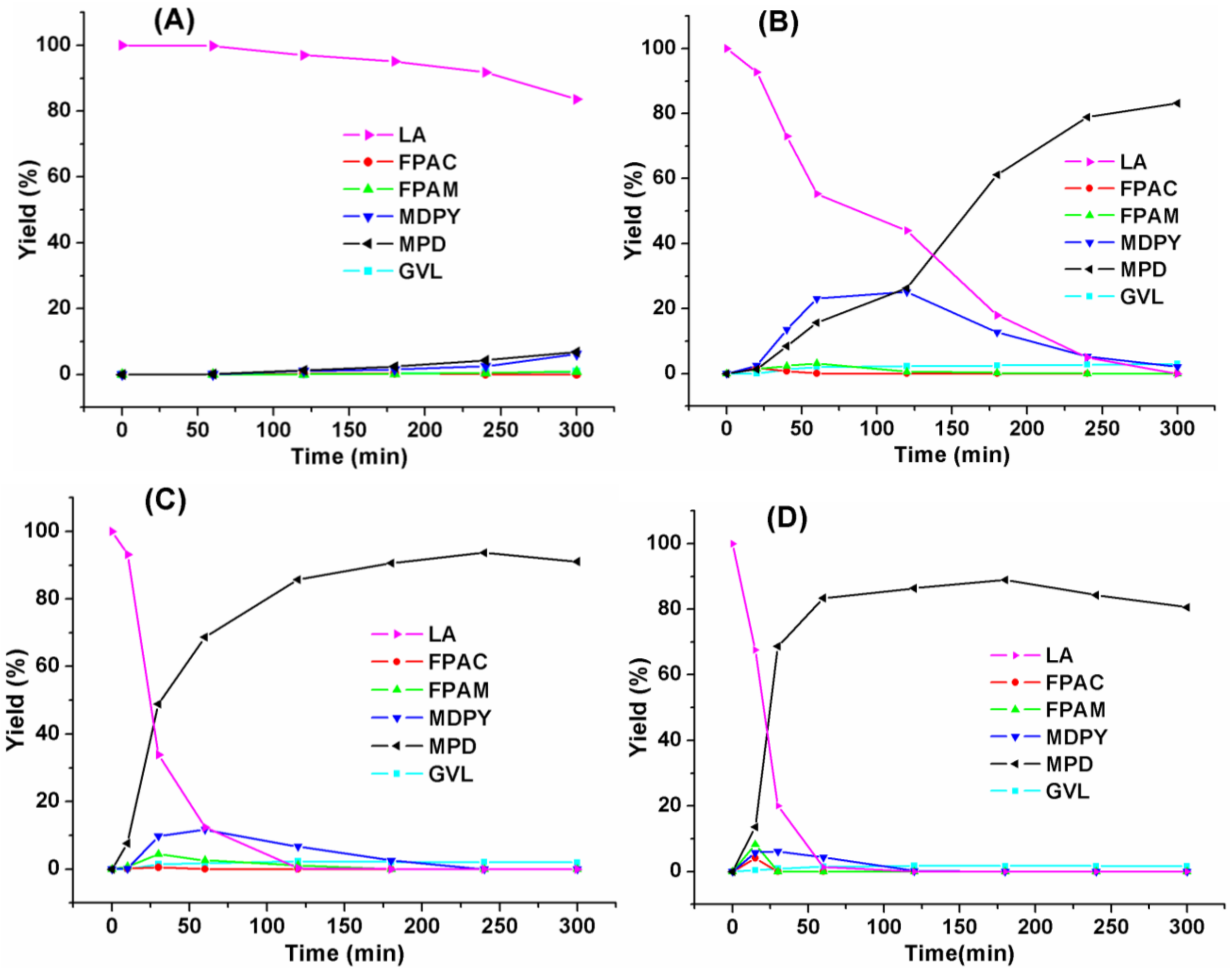

Figure S3. Effect of reaction temperature and time on the synthesis of MPD from LA at (A) 120 °C, (B) 140 °C, (C) 160°C, and (D) 180 °C.
Conditions: 2 mmol LA, 6 equiv. H_2NCHO, 30 equiv. H_2O.

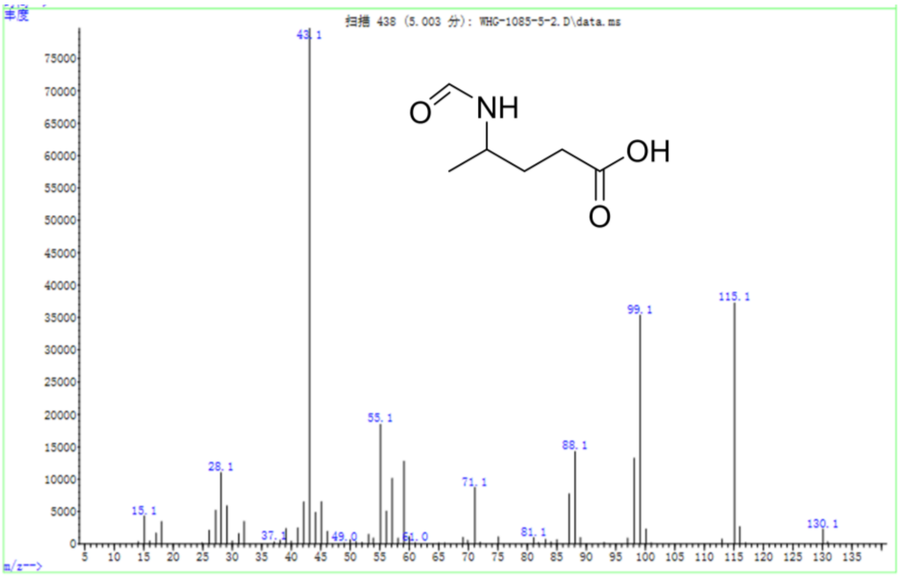

丰度
扫描 438 (5.003 分): WHG-1085-5-2.D\data.ms
m/z-->
43.1
99.1
115.1
55.1
88.1
28.1
71.1
15.1
130.1
O
NH
OH
O

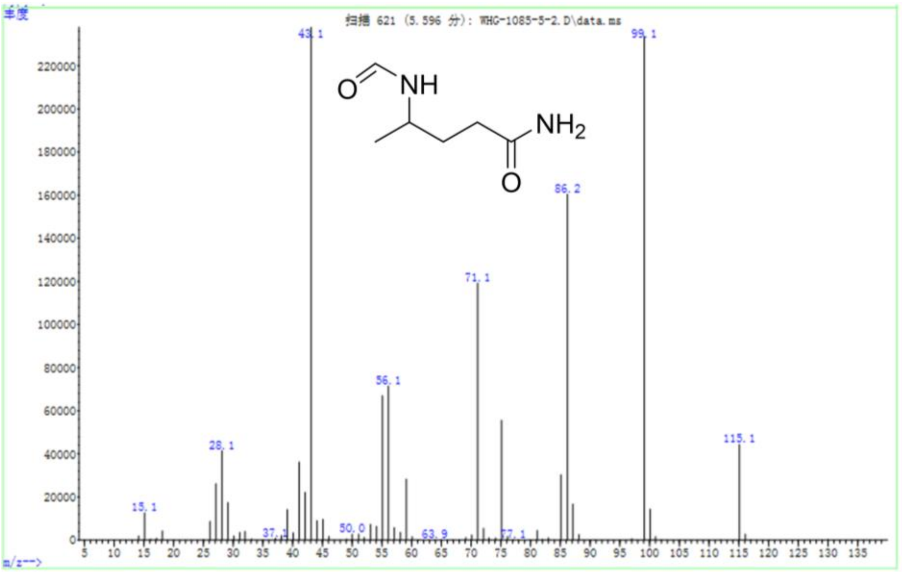

丰度
扫描 621 (5.596 分): WHG-1085-5-2.D\data.ms
m/z-->
43.1
99.1
86.2
71.1
56.1
115.1
28.1
15.1
O
NH
NH2
O

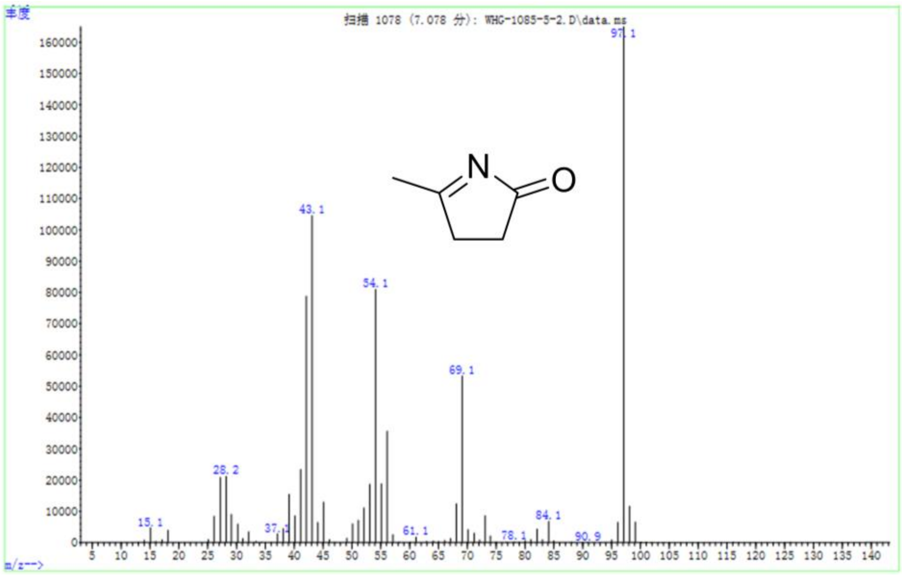

丰度
扫描 1078 (7.078 分): WHG-1085-5-2.D\data.ms
m/z-->
97.1
43.1
54.1
69.1
28.2
15.1
84.1
N
O

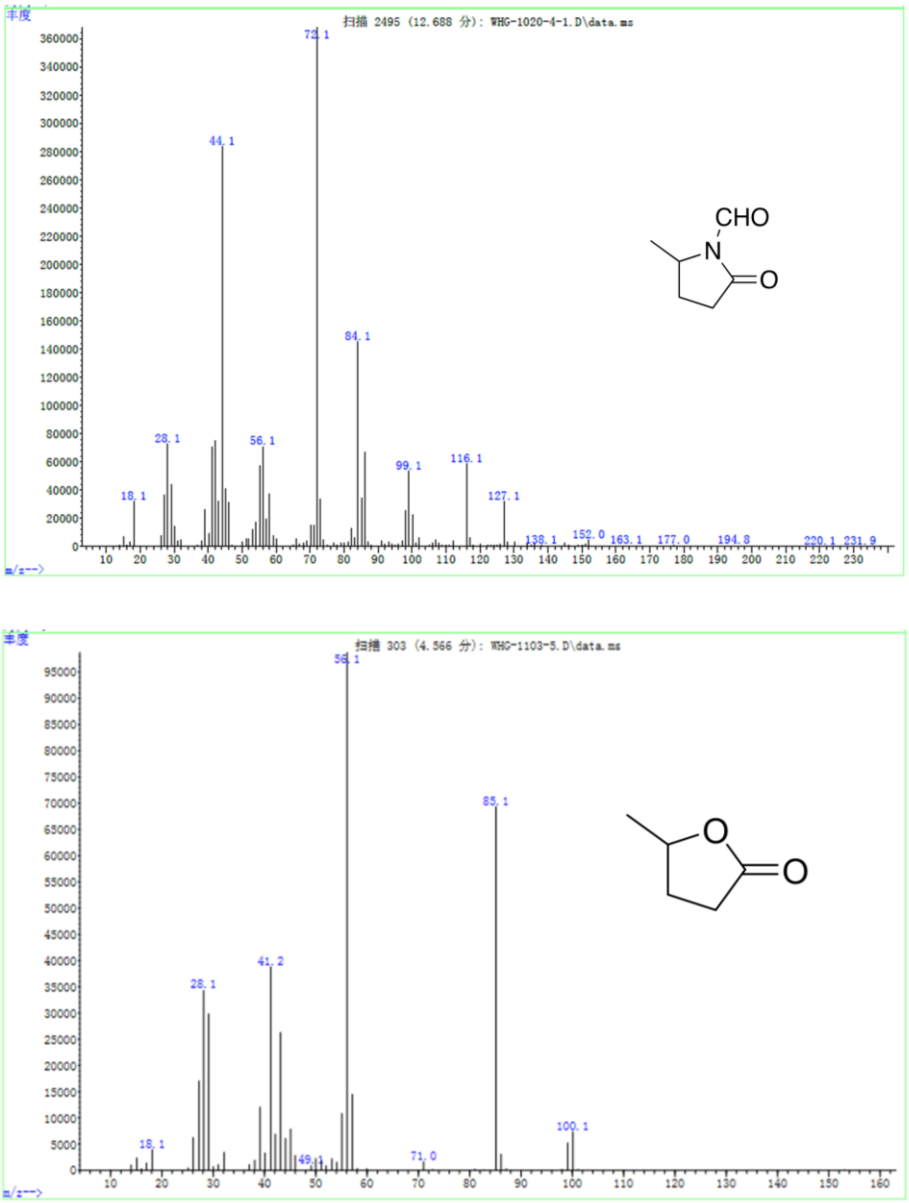

Figure S4. GC-MS spectra of dominant co-products formed in the LA-to-MPD reaction systems.

In view of the complexity of this reaction system, *in situ* ATR-IR spectra of the reaction mixture containing LA, H_2NCHO and water heated at 160 °C for varying times were recorded to trace the real-time products (Figure S5). As expected, H_2NCHO (ν_{C-H}/$\nu_{C=O}$: ~2600 cm^{-1}; $\nu_{HC=O}$: 1450 cm^{-1}; ν_{C-N}: 1320 cm^{-1}; ν_{N-H}: 710 cm^{-1}) is not completely decomposed and detected by IR spectroscopy throughout the reaction. In addition to the characteristic absorption bands belonging to GVL (ν_{C-O}: ~990 cm^{-1}) and MPD ($\nu_{C=O}$: ~1410 cm^{-1}; ν_{C-N}: 1240 cm^{-1}; ν_{N-H}: 940 cm^{-1}), a cyclic imide ($\nu_{-C=N}$: ~2050 cm^{-1}), assignable to MDPY, is observed. The initial increase and later decrease of the band intensity support the intermediate character of MDPY and agrees with the data in Figure S5. HCOOH cannot be detected in this case due to the overlapping bands with other molecules like water and H_2NCHO.

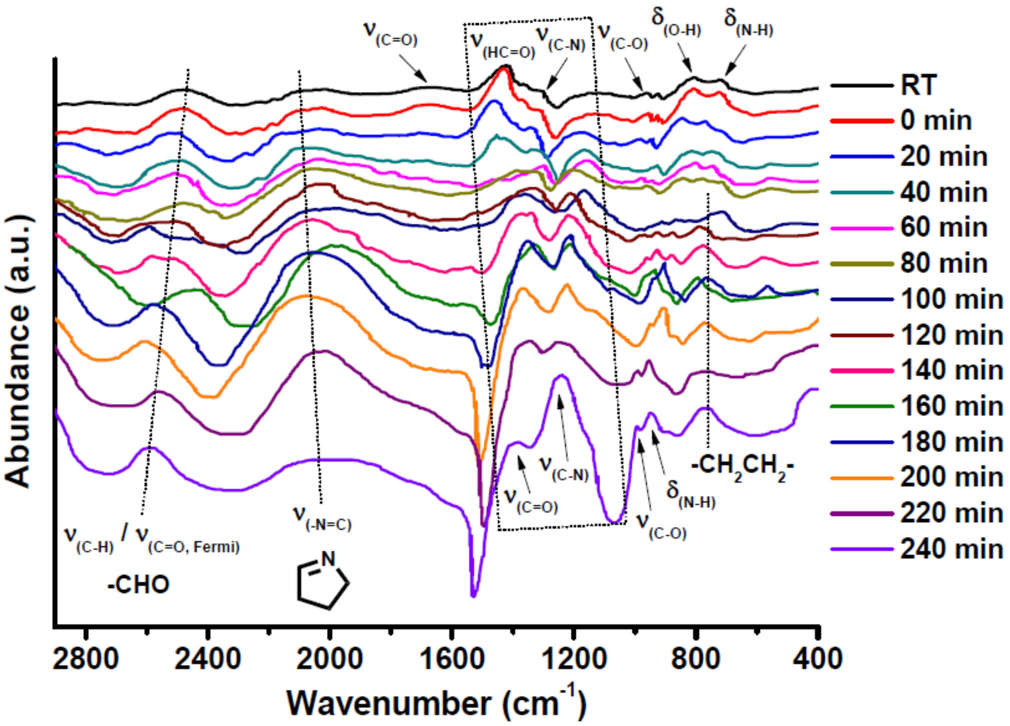

Figure S5. *In situ* ATR-IR spectra of the reaction mixture containing LA, H_2NCHO and water under static conditions heated at 160 °C for variable times.

Note: water presented in the system shifts the product wavenumber somehow.

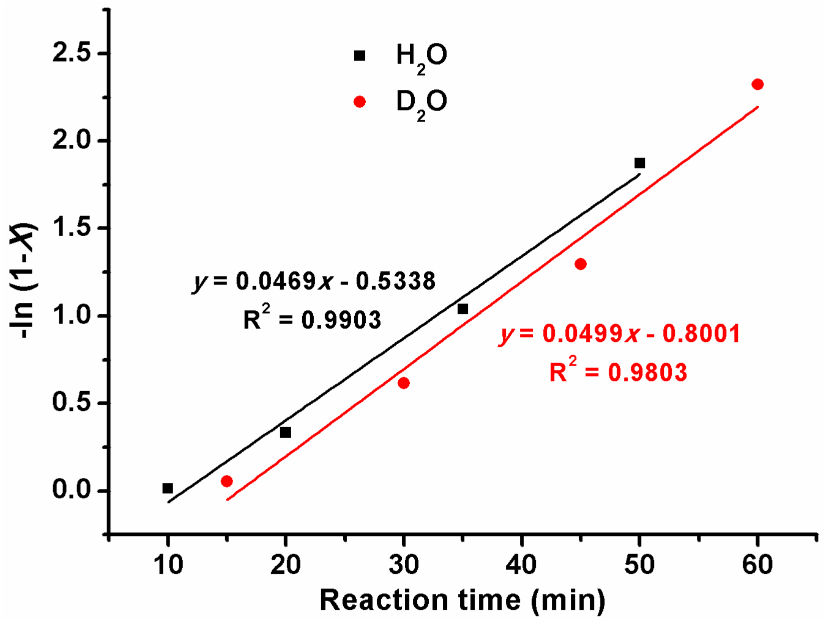

Figure S6. Kinetic profiles for conversion of LA to MPD with normal and deuterium water. Reaction conditions: 2 mmol LA, 6 equiv. H_2NCHO, 30 equiv. water, 160 °C, 10-60 min.

Assumed that LA-to-MPD conversion is a 1st order process, the reaction rate could be expressed as follows: d[MPD]/dt = k[LA] = d[LA]/dt; after the subsequent integral calculation, the original equation would further become: -ln(1 - X) = kt + C. Values of -ln(1 - X) (where X is conversion of LA) were plotted against reaction time (t) at different temperatures in order to obtain rate constants (k). The good linear correlation between -ln(1 - X) and t indicates the pseudo first order reaction. In this regard, it can be found from Figure S6 that k_H = 0.0469 min^{-1}, k_D = 0.0499 min^{-1}; **k_H/k_D = 0.94**.

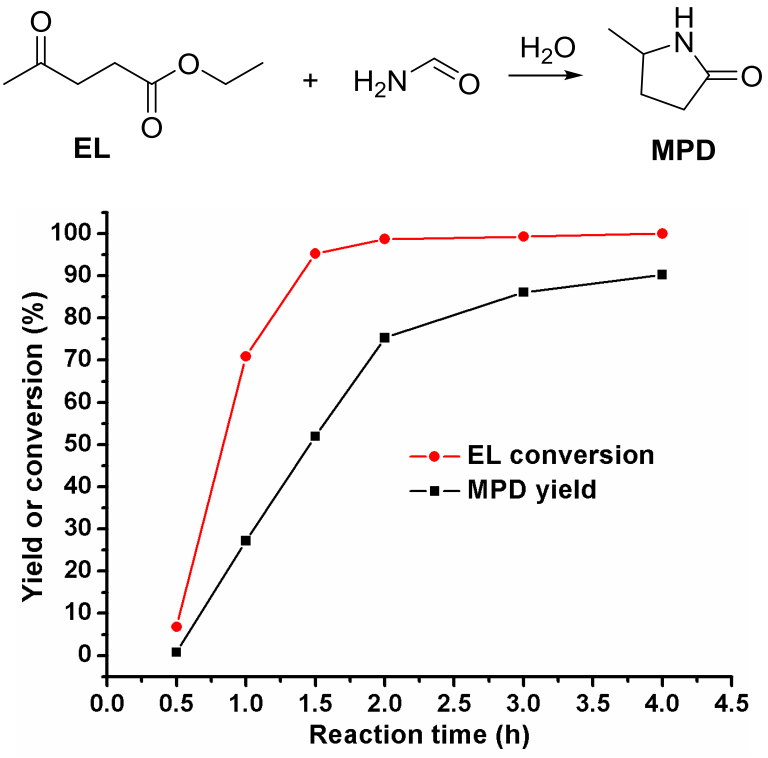

Figure S7. Water-enabled cycloamination of EL with H_2NCHO to MPD
Reaction conditions: 2 mmol EL, 6 equiv. H_2NCHO, 30 equiv. H_2O, 160 °C, 0.5-4 h.

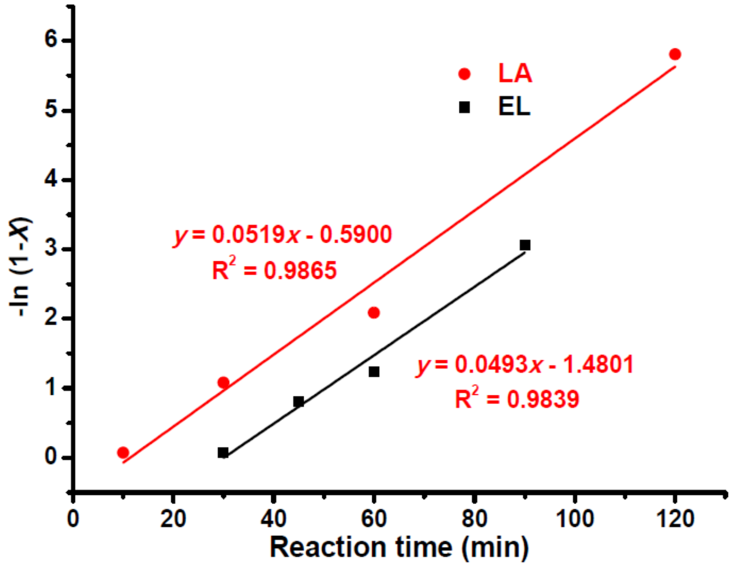

Figure S8. Kinetic profiles for conversion of LA or EL to MPD
Reaction conditions: 2 mmol LA or EL, 6 equiv. H_2NCHO, 30 equiv. water, 160 ºC, 10-60 min.

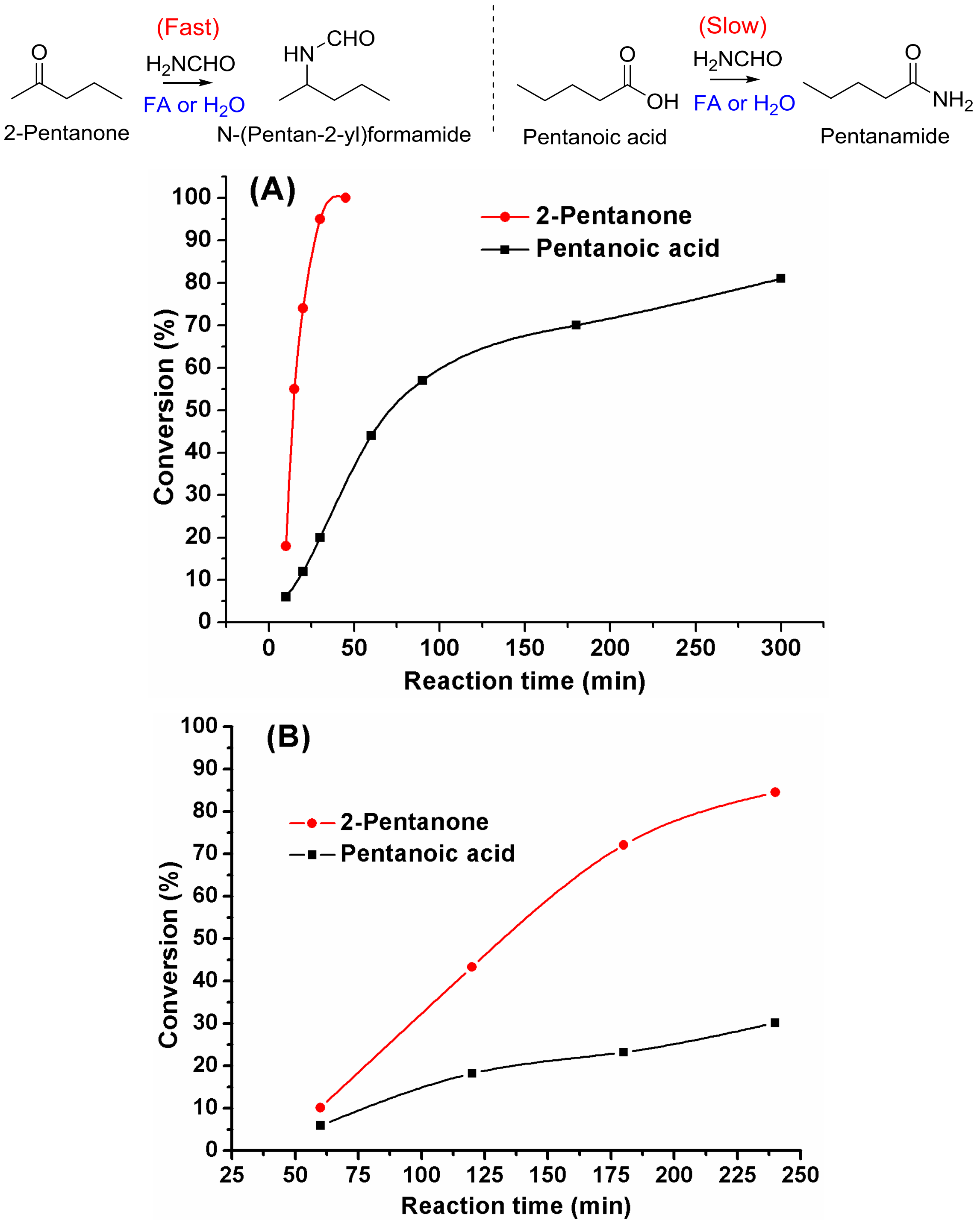

Figure S9. Reaction kinetics for conversion of 2-pentanone and pentanoic acid with H_2NCHO and HCOOH (A), or with H_2NCHO and water (B).

Reaction conditions: (A) 2 mmol 2-pentanone or pentanoic acid, 6 mmol HCOOH, 20 mmol H_2NCHO, 160 °C, 10-300 min; (B) 2 mmol 2-pentanone or pentanoic acid, 6 equiv. H_2NCHO, 30 equiv. water, 160 °C, 60-240 min.

To elaborate the occurrence of hydrogen transfer with *in situ* formed HCOOH, several deuterium-labeled experiments were conducted (Figures S10-S12). Starting from LA and H_2NCHO with D_2O, the molecular ion peak of obtained MPD was found to have additional 2-5 amu (Figure S10), indicating that the H/D exchange concurrently takes place along with hydrogen transfer process. When 2-pentanone or pentanoic acid instead of LA was employed to react with H_2NCHO and D_2O under identical conditions, only additional 1-2 amu was observed in the resulting product *N*-(pentan-2-yl)formamide or pentanamide, respectively (Figures S11 & S12). These results on the other hand demonstrate the presence of ALs and unsaturated cycloamines with resonating structures of MDPY, wherein more deuterium atoms can be incorporated in the LA cycloamination processes.

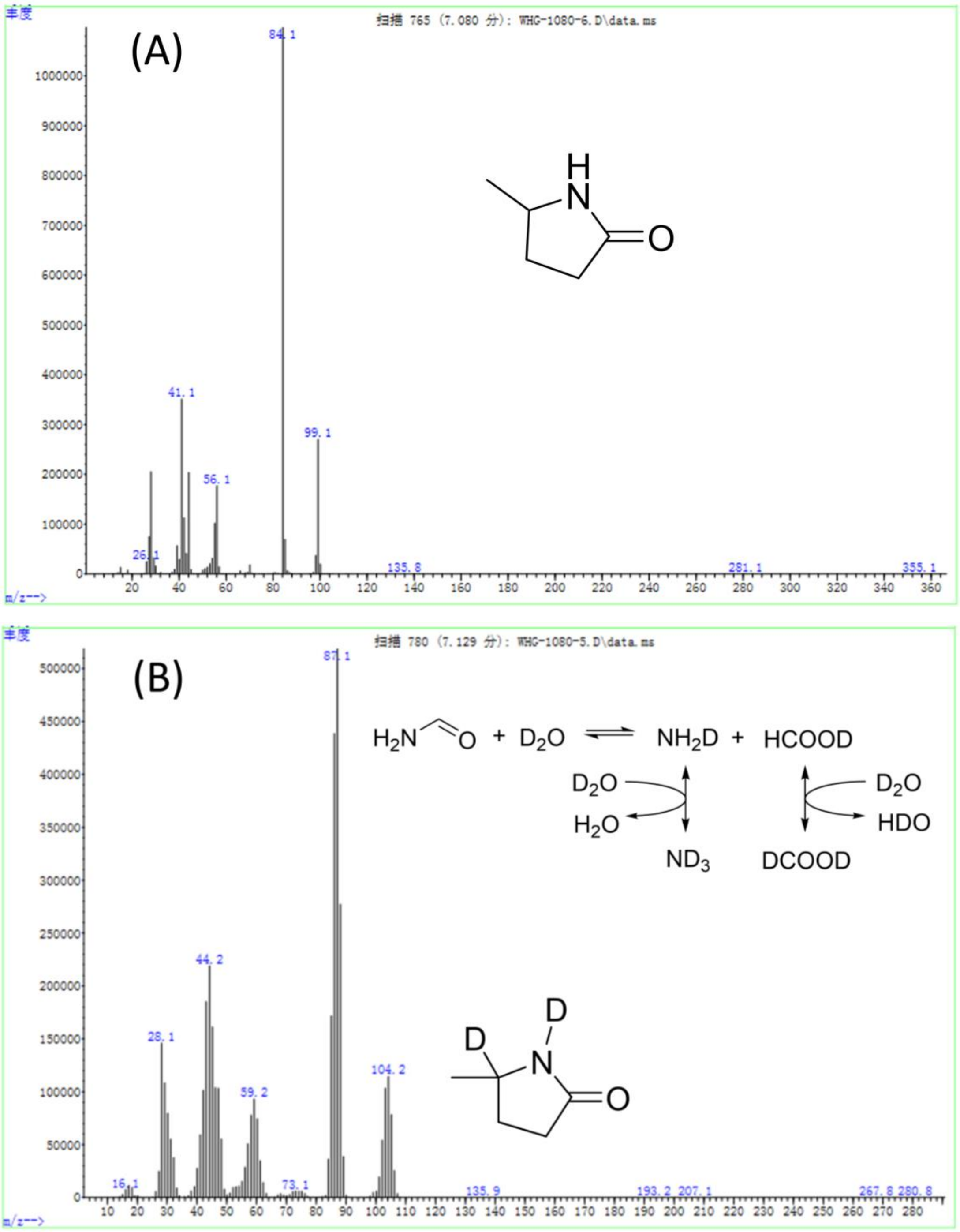

Figure S10. GC-MS spectra of MPD obtained from LA and H_2NCHO with (A) normal and (B) deuterium-labeled water.

Reaction conditions: 2 mmol LA, 6 equiv. H_2NCHO, 30 equiv. water, 160 ºC, 4 h.

Additional m/z observed in Figure S10 B for the product (MPD) is resulted from the incorporation of more deuterium into the ALs and MDPY tautomeric structures via the ion-exchange and hydrogen transfer processes.

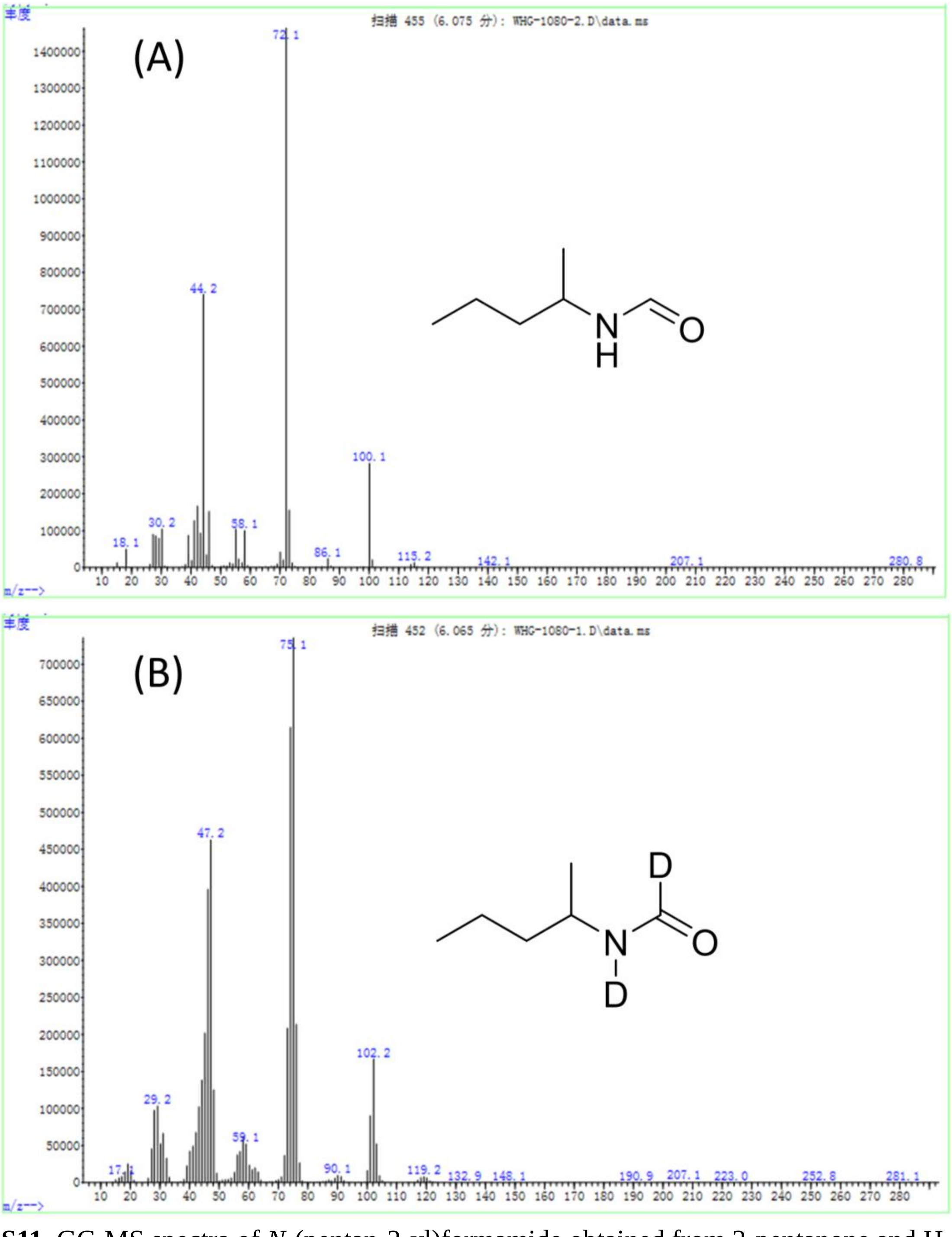

Figure S11. GC-MS spectra of *N*-(pentan-2-yl)formamide obtained from 2-pentanone and H_2NCHO with (A) normal and (B) deuterium-labeled water.

Reaction conditions: 2 mmol 2-pentanone, 6 equiv. H_2NCHO, 30 equiv. water, 160 ºC, 4 h.

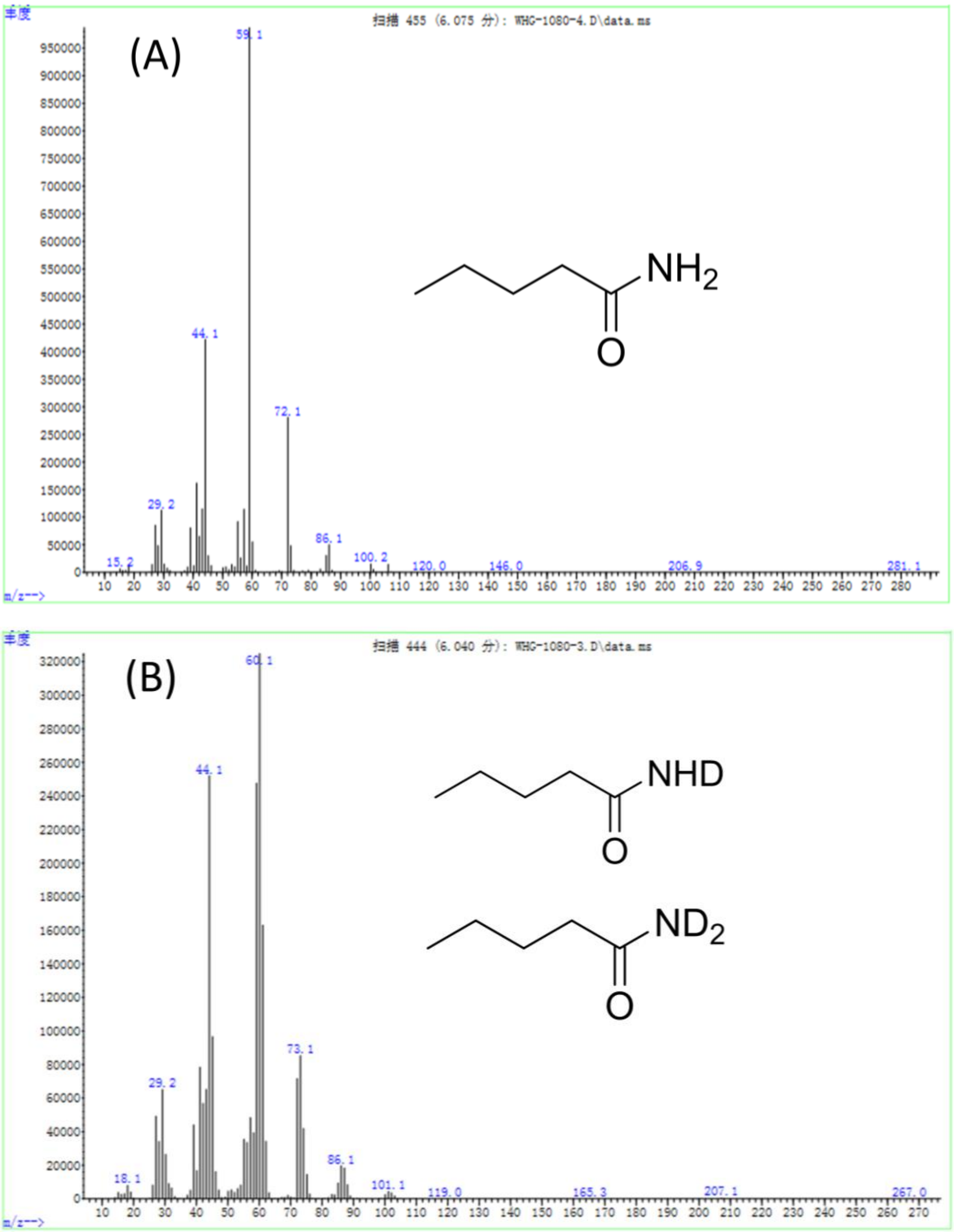

Figure S12. GC-MS spectra of pentanamide obtained from pentanoic acid and AM with (A) normal and (B) deuterium-labeled water.

Reaction conditions: 2 mmol pentanoic acid, 6 equiv. AM, 30 equiv. water, 160 ºC, 4 h.

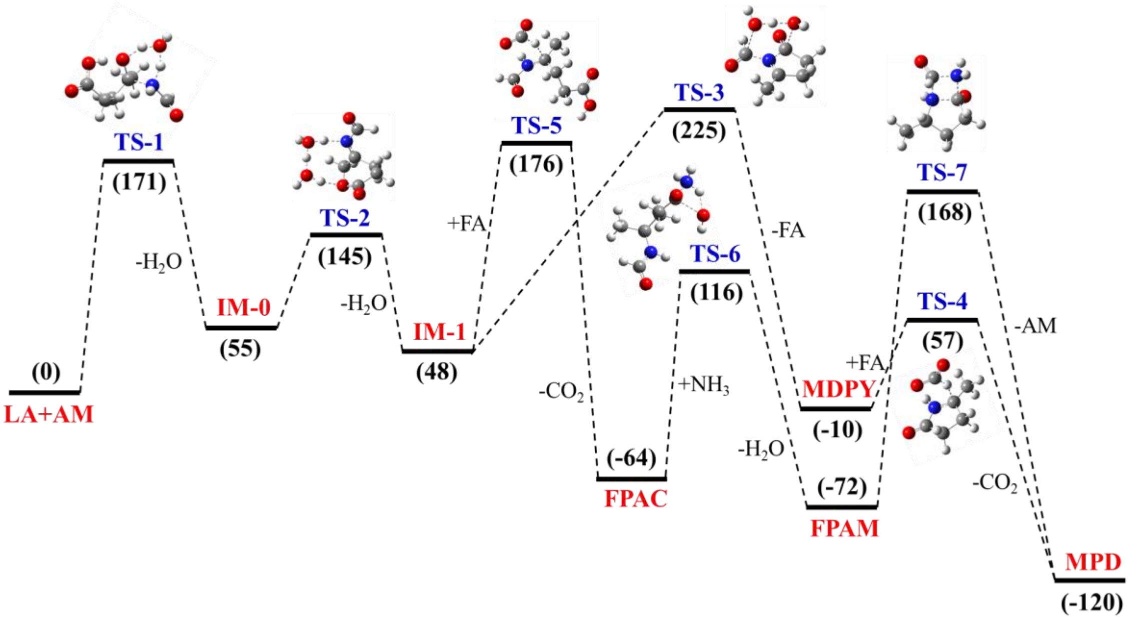

Figure S13. Computational free energy diagrams for reaction pathways of LA-to-MPD conversion. Values in parentheses are computational free energies (kJ mol^{-1}) with respect to LA and H_2NCHO. (AM: H_2NCHO, FA: HCOOH)

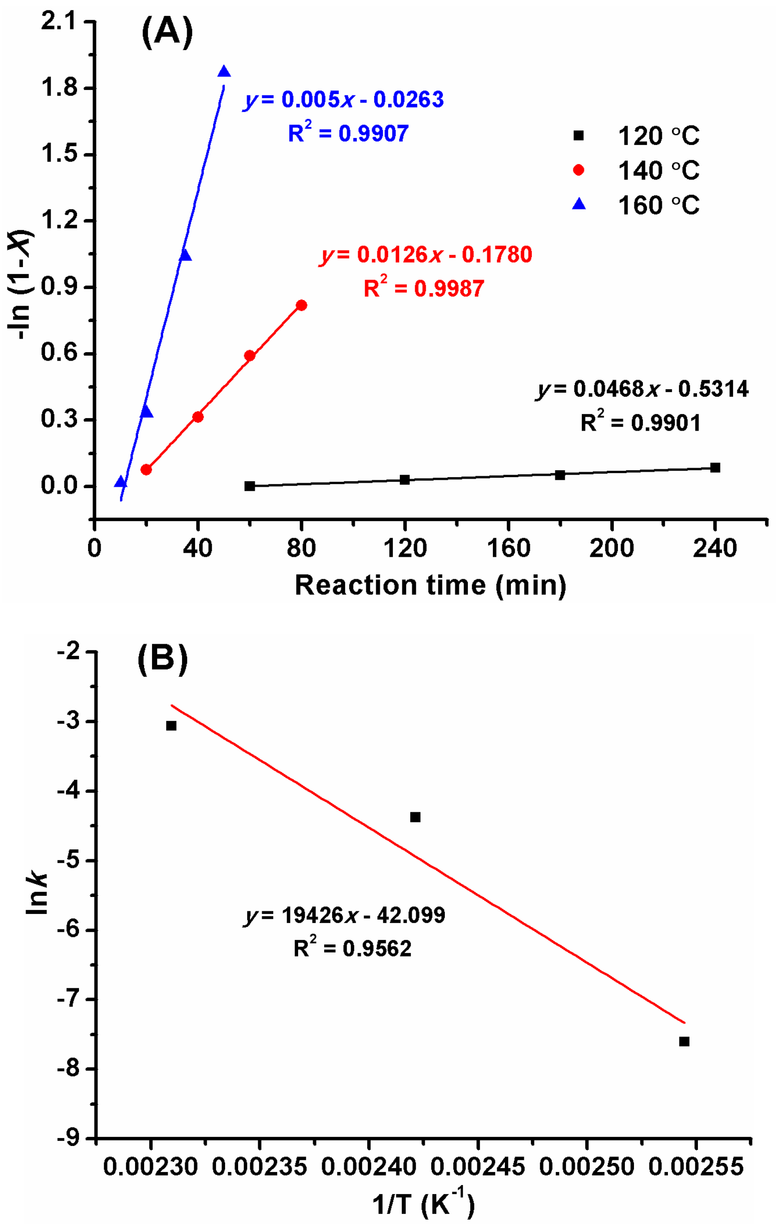

Figure S14. (A) Kinetic profiles and (B) Arrhenius plot for conversion of LA to MPD. Reaction conditions: 2 mmol LA, 6 equiv. H_2NCHO, and 30 equiv. H_2O.

Assumed that LA-to-MPD conversion is a 1st order process, the reaction rate could be expressed as follows: d[MPD]/d*t* = k[LA] = d[LA]/d*t*; after the subsequent integral calculation, the original equation would further become: -ln(1 - *X*) = *k*t + C. Values of -ln(1 - *X*) (where *X* is conversion of LA) were plotted against reaction time (*t*) at different temperatures in order to obtain rate constants (*k*). Then the activation energy (Ea) was calculated from rate constants by the Arrhenius equation: ln*k* = -Ea/RT + lnA, and found to be **<u>162 kJ mol^{-1}</u>**.

Characterization data of products

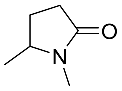

[S21]

^{1}H NMR (400 MHz, $CDCl_3$) δ ppm: 3.50 – 3.57 (m, 1H), 2.74 (s, 3H), 2.10 – 2.43 (m, 3H), 1.47 – 1.59 (m, 1H), 1.17 (d, *J* = 6.3 Hz, 3H). ^{13}C NMR (100 MHz, $CDCl_3$) δ ppm: 174.91, 55.62, 30.23, 27.34, 26.65, 19.76. HRMS: Calculated for $C_6H_{11}NO$, [M+H] 114.0841, found 114.0792.

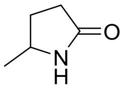

[S22]

^{1}H NMR (600 MHz, DMSO-d_6) δ ppm: 7.62 (s, 1H), 3.60 (dd, *J* = 12.7, 6.4 Hz, 1H), 2.15 – 2.09 (m, 3H), 1.51 – 1.46 (m, 1H), 1.09 (d, *J* = 6.2 Hz, 3H). ^{13}C NMR (151 MHz, DMSO-d_6) δ ppm: 176.80, 49.52, 30.76, 29.21, 22.61. HRMS: Calculated for C_5H_9NO, [M+H] 100.0684, found 100.0678.

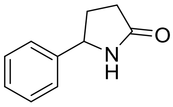

[S23]

^{1}H NMR (500 MHz, $CDCl_3$) δ ppm: 7.35 (t, *J* = 7.7 Hz, 2H), 7.28 (t, *J* = 6.7 Hz, 3H), 6.54 (s, 1H), 4.74 (t, *J* = 7.1 Hz, 1H), 2.60 – 2.51 (m, 1H), 2.49 – 2.36 (m, 2H), 2.00 – 1.91 (m, 1H). ^{13}C NMR (126 MHz, $CDCl_3$) δ ppm: 178.89, 142.54, 129.00, 128.01, 125.73, 58.25, 31.45, 30.45. HRMS: Calculated for $C_{10}H_{11}NO$, [M+H] 162.0841, found 162.0836.

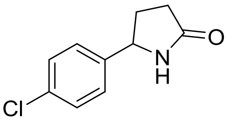

[S23]

^{1}H NMR (500 MHz, $CDCl_3$) δ ppm: 7.33 (d, *J* = 8.5 Hz, 2H), 7.22 (d, *J* = 8.4 Hz, 2H), 6.55 (s, 1H), 4.73 (t, *J* = 7.1 Hz, 1H), 2.60 – 2.52 (m, 1H), 2.49 – 2.35 (m, 2H), 1.95 – 1.87 (m, 1H). ^{13}C NMR (126 MHz, $CDCl_3$) δ ppm: 178.73, 141.08, 133.70, 129.18, 127.12, 57.62, 31.43, 30.32. HRMS: Calculated for $C_{10}H_{10}ClNO$, [M+H] 196.0451, found 196.0447.

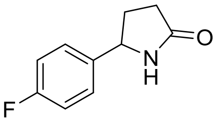

[S23]

^{1}H NMR (500 MHz, $CDCl_3$) δ ppm: 7.26 (t, *J* = 7.0 Hz, 2H), 7.04 (t, *J* = 8.6 Hz, 2H), 6.54 (s, 1H), 4.74 (t, *J* = 7.2 Hz, 1H), 2.59 – 2.51 (m, 1H), 2.49 – 2.36 (m, 2H), 1.96 – 1.87 (m, 1H). ^{13}C NMR (126 MHz, $CDCl_3$) δ ppm: 178.70, 162.43, 138.27, 127.40, 115.89, 57.63, 31.57, 30.41. HRMS: Calculated for $C_{10}H_{10}FNO$, [M+H] 180.0746, found 180.0741.

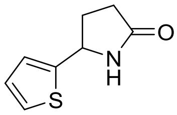

[S24]

^{1}H NMR (500 MHz, $CDCl_3$) δ ppm: 7.24 (dd, *J* = 5.0, 1.3 Hz, 1H), 6.97 (dd, *J* = 4.2, 1.2 Hz, 1H), 6.95 (dd, *J* = 5.0, 3.5 Hz, 1H), 6.69 (s, 1H), 5.04 – 4.96 (m, 1H), 2.63 – 2.55 (m, 1H), 2.54 – 2.35 (m, 2H), 2.15 – 2.07 (m, 1H). ^{13}C NMR (126 MHz, $CDCl_3$) δ ppm: 178.03 (s), 146.54 (s), 126.89 (s), 125.07 (s), 124.24 (s), 54.02 (s), 31.75 (s), 30.23 (s). HRMS: Calculated for C_8H_9NOS, [M+H] 168.0405, found 168.0400.

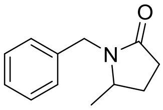

[S22]

^{1}H NMR (400 MHz, $CDCl_3$) δ ppm: 7.33 – 7.22 (m, 5H), 4.95 (d, *J* = 14.0 Hz, 1H), 3.99 (d, *J* = 16.0 Hz, 1H), 3.56 – 3.49 (m, 1H), 2.48 – 2.34 (m, 2H), 2.18 – 2.11 (m, 1H), 1.63 – 1.53 (m, 1H), 1.14 (d, *J* = 8.0 Hz, 3H). ^{13}C NMR (100 MHz, $CDCl_3$) δ ppm: 175.01 (s), 136.82 (s), 128.63 (s), 128.04 (s), 127.45 (s), 52.91 (s), 44.02 (s), 30.31 (s), 26.72 (s), 19.63 (s). HRMS: Calculated for $C_{12}H_{15}NO$, [M+H] 190.1154, found: 190.1148.

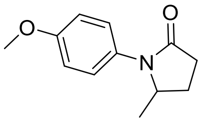

[S22]

^{1}H NMR (400 MHz, $CDCl_3$) δ ppm: 7.18 (d, *J* = 8.0 Hz, 1H), 7.16 – 7.13 (m, 1H), 6.86 (d, *J* = 8.0 Hz, 1H), 6.84 (d, *J* = 4.0 Hz, 1H), 4.15 – 4.07 (m, 1H), 3.73 (s, 3H), 2.58 – 2.41 (m, 2H), 2.33 – 2.25 (m, 1H), 1.71 – 1.62 (m, 1H), 1.10 (d, *J* = 8.0 Hz, 3H). ^{13}C NMR (100 MHz, $CDCl_3$) δ ppm: 174.32 (s), 157.71 (s), 130.43 (s), 126.12 (s), 114.43 (s), 56.14 (s), 55.45 (s), 31.12 (s), 26.83 (s), 20.33 (s). HRMS: Calculated for $C_{12}H_{15}NO_2$, [M+H] 206.1103, found: 206.1097.

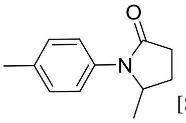

[S22]

^{1}H NMR (400 MHz, $CDCl_3$) δ ppm: 7.23 (d, *J* = 8.4 Hz, 2H), 7.18 (d, *J* = 8.4 Hz, 2H), 4.24(dd, *J* =13.0, 6.0 Hz, 1H), 2.66 – 2.45 (m, 2H), 2.40 – 2.34 (m, 4H), 1.78 – 1.71 (m, 1H), 1.19 (d, *J* = 6.0 Hz, 3H). ^{13}C NMR (100 MHz, $CDCl_3$) δ ppm: 174.21, 135.62, 135.03, 129.64, 124.25, 55.86, 31.37, 26.82, 21.01, 20.23. HRMS: Calculated for $C_{12}H_{15}NO$, [M+H] 190.1154, found: 190.1156.

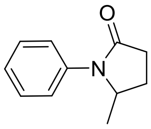

[S22]

^{1}H NMR (400 MHz, $CDCl_3$) δ ppm: 7.41 – 7.33 (m, 4H), 7.24 – 7.19 (m, 1H), 4.34 – 4.26 (m, 1H), 2.68 – 2.50 (m, 2H), 2.42 – 2.33 (m, 1H), 1.80 – 1.72 (m, 1H), 1.21 (d, *J* = 8.0 Hz, 3H). ^{13}C NMR (100 MHz, $CDCl_3$) δ ppm: 174.22 (s), 137.61 (s), 129.02 (s), 125.73 (s), 124.03 (s), 55.60 (s), 31.33

(s), 26.81 (s), 20.21 (s). HRMS: Calculated for $C_{11}H_{13}NO$, [M+H] 176.0997, found: 176.0992.

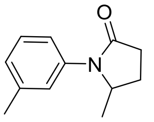

^{1}H NMR (400 MHz, $CDCl_3$) δ ppm: 7.19 (t, *J* = 7.7 Hz, 1H), 7.12 (s, 1H), 7.05 (d, *J* = 7.8 Hz, 1H), 6.94 (d, *J* = 7.4 Hz, 1H), 4.18 (dd, *J* = 12.7, 6.3 Hz, 1H), 2.64 – 2.38 (m, 2H), 2.37 – 2.21 (m, 4H), 1.66 (ddd, *J* = 13.0, 11.0, 7.2 Hz, 1H), 1.11 (d, *J* = 6.2 Hz, 3H). ^{13}C NMR (101 MHz, $CDCl_3$) δ ppm: 174.11 (s), 138.75 (s), 137.40 (s), 128.69 (s), 126.62 (s), 124.98 (s), 121.14 (s), 55.69 (s), 31.26 (s), 26.70 (s), 21.40 (s), 20.14 (s). HRMS: Calculated for $C_{12}H_{15}NO$, [M+H] 190.1154, Found: 190.1149.

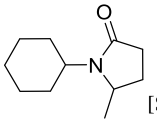
[S21]

^{1}H NMR (400 MHz, $CDCl_3$) δ ppm: 3.86 – 3.60 (m, 2H), 2.50 – 2.40 (m, 1H), 2.32 – 2.20 (m, 1H), 2.19 – 2.11 (m, 1H), 1.84 – 1.75 (m, 3H), 1.68 – 1.46 (m, 5H), 1.43 – 1.24 (m, 2H), 1.21 (d, *J* = 4.0 Hz, 2H), 1.17 – 1.10 (m, 1H). ^{13}C NMR (100 MHz, $CDCl_3$) δ ppm: 174.51 (s), 53.02 (s), 52.63 (s), 31.84 (s), 30.35 (s), 30.16 (s), 27.53 (s), 26.04 (s), 25.91 (s), 25.63 (s), 22.34 (s). HRMS: Calculated for $C_{11}H_{19}NO$, [M+H] 182.1467, Found: 182.1463.

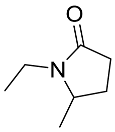
[S21]

^{1}H NMR (400 MHz, $CDCl_3$) δ ppm: 3.52 – 3.42 (m, 1H), 3.34 (m, 2H), 2.31-2.13 (m, 2H), 2.07 – 1.89 (m, 1H), 1.81 – 1.72 (m, 1H), 1.27 (d, 3H), 1.15 (t, 3H); ^{13}C NMR (100 MHz, $CDCl_3$) δ ppm: 175.23, 53.51, 71.62, 30.23, 26.81, 20.84, 20.16. HRMS: Calculated for $C_7H_{13}NO$, [M+H] 128.0997, Found: 128.1021.

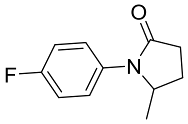
[S25]

^{1}H NMR (400 MHz, $CDCl_3$) δ ppm: 7.33 – 7.30 (m, 2H), 7.10 – 7.06 (m, 2H), 4.23(dd, *J* = 12.9, 6.4 Hz, 1H), 2.67 – 2.49 (m, 2H), 2.42 – 2.33 (m, 1H), 1.80 – 1.73 (m, 1H), 1.19 (d, *J* = 6.0 Hz, 3H); ^{13}C NMR (100 MHz, $CDCl_3$) δ ppm: 174.23, 160.41, 133.62, 126.03, 115.81, 55.84, 31.16, 26.82, 20.14. HRMS: Calculated for $C_{11}H_{12}FNO$, [M+H] 194.0903, Found: 194.0901.

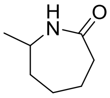

^{1}H NMR (300 MHz, $CDCl_3$) δ ppm: 1.23 (d, *J* = 3.00 Hz, 3H), 1.33 – 1.38 (m, 2H), 1.56 – 1.65 (m, 2H), 2.31 (t, *J* = 6.00Hz, 1H), 3.41 – 3.48 (m, 1H). ^{13}C NMR (75 MHz, $CDCl_3$) δ ppm: 173.81, 92.72,

35.83, 33.84, 25.55, 24.74, 19.82. HRMS: Calculated for $C_7H_{13}NO$, [M+H] 128.0997, Found: 128.0989.

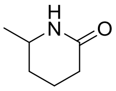

^{1}H NMR (300 MHz, $CDCl_3$) δ ppm: 0.89 (t, *J* = 6.00 Hz, 3H), δ 1.16 (d, *J* =3.00 Hz, 3H), 1.62 – 1.67 (m, 2H), 1.82 – 1.87 (m, 2H), 2.22 – 2.31 (m, 1H), 3.44-3.48 (m, 1H). ^{13}C NMR (75 MHz, $CDCl_3$) δ ppm: 172.51, 48.72, 30.93, 30.34, 22.63, 19.74. HRMS: Calculated for $C_6H_{11}NO$, [M+H] 114.0841, Found: 114.0901.

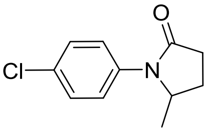

[S22]

^{1}H NMR (400 MHz, $CDCl_3$) δ ppm: 7.35 (s, 4H), 4.32 – 4.24 (dd, *J* = 12.9, 6.4 Hz, 1H), 2.68 – 2.49 (m, 2H), 2.42 – 2.33 (m, 1H), 1.80 – 1.71 (m, 1H), 1.21 (d, *J* = 8.0 Hz, 3H). ^{13}C NMR (100 MHz, $CDCl_3$) δ ppm: 174.21, 136.23, 130.92, 129.04, 124.93, 55.45, 31.24, 26.64, 20.03. HRMS: Calculated for $C_{11}H_{12}ClNO$, [M+H] 210.0607, found 210.0598.

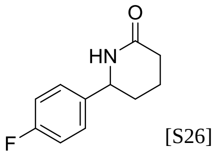

[S26]

^{1}H NMR (500 MHz, $CDCl_3$) δ ppm: 7.25 (dd, *J* = 8.8, 5.0 Hz, 2H), 7.04 (t, *J* = 8.6 Hz, 2H), 6.07 (s, 1H), 4.53 (dd, *J* = 9.1, 4.5 Hz, 1H), 2.50 – 2.36 (m, 2H), 2.11 – 2.04 (m, 1H), 1.92 – 1.86 (m, 1H), 1.82 – 1.73 (m, 1H), 1.66 – 1.58 (m, 1H). ^{13}C NMR (126 MHz, $CDCl_3$) δ ppm: 172.50, 162.40, 138.35, 127.84, 115.82, 57.24, 32.36, 31.32, 19.70. HRMS: Calculated for $C_{11}H_{12}PNO$, [M+H] 194.0903, found 194.0897.

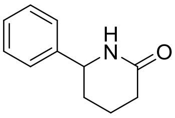

[S26]

^{1}H NMR (500 MHz, $CDCl_3$) δ ppm: 7.37 – 7.33 (m, 2H), 7.29 – 7.26 (m, 3H), 6.00 (s, 1H), 4.53 (dd, *J* = 9.0, 4.6 Hz, 1H), 2.50 – 2.36 (m, 2H), 2.12 – 2.06 (m, 1H), 1.93 – 1.86 (m, 2H), 1.82 – 1.73 (m, 1H), 1.70 – 1.61 (m, 1H). ^{13}C NMR (126 MHz, $CDCl_3$) δ ppm: 172.52, 142.60, 128.93, 128.04, 126.17, 94.48, 57.86, 32.26, 31.36, 19.75. HRMS: Calculated for $C_{11}H_{13}NO$, [M+H] 176.0997, found 176.0986.

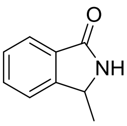

[S27]

^{1}H NMR (500 MHz, $CDCl_3$) δ ppm: 8.25 (s, 1H), 7.86 (d, *J* = 8.2 Hz, 1H), 7.56 (t, *J* = 8.0 Hz, 1H), 7.49 – 7.45 (m, 2H), 4.47 (s, 1H), 1.56 (d, *J* = 6.7 Hz, 3H). ^{13}C NMR (126 MHz, $CDCl_3$) δ ppm: 172.49, 143.83, 132.34, 131.80, 128.07, 123.72 123.29, 52.24, 20.35. HRMS: Calculated for C_9H_9NO, [M+H] 148.0684, found 148.0679.

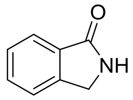

[S27]

^{1}H NMR (500 MHz, $CDCl_3$) δ ppm: 8.27 (s, 1H), 7.85 (d, *J* = 8.2 Hz, 1H), 7.57 (t, *J* = 8.0 Hz, 1H), 7.50 – 7.46 (m, 2H), 4.48 (s, 2H). ^{13}C NMR (126 MHz, $CDCl_3$) δ ppm: 172.47, 143.82, 132.32, 131.79, 128.06, 123.71, 123.30, 45.89. HRMS: Calculated for C_8H_7NO, [M+H] 134.0528, found 134.0517.

NMR spectra of products

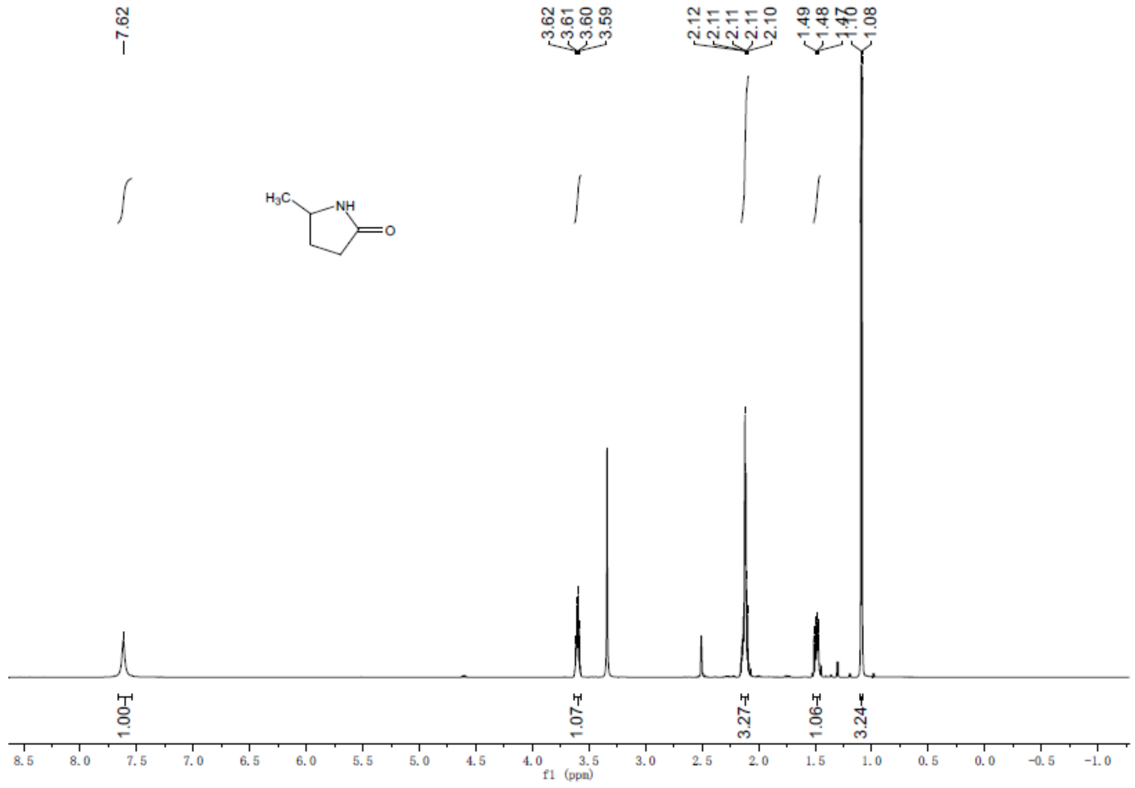

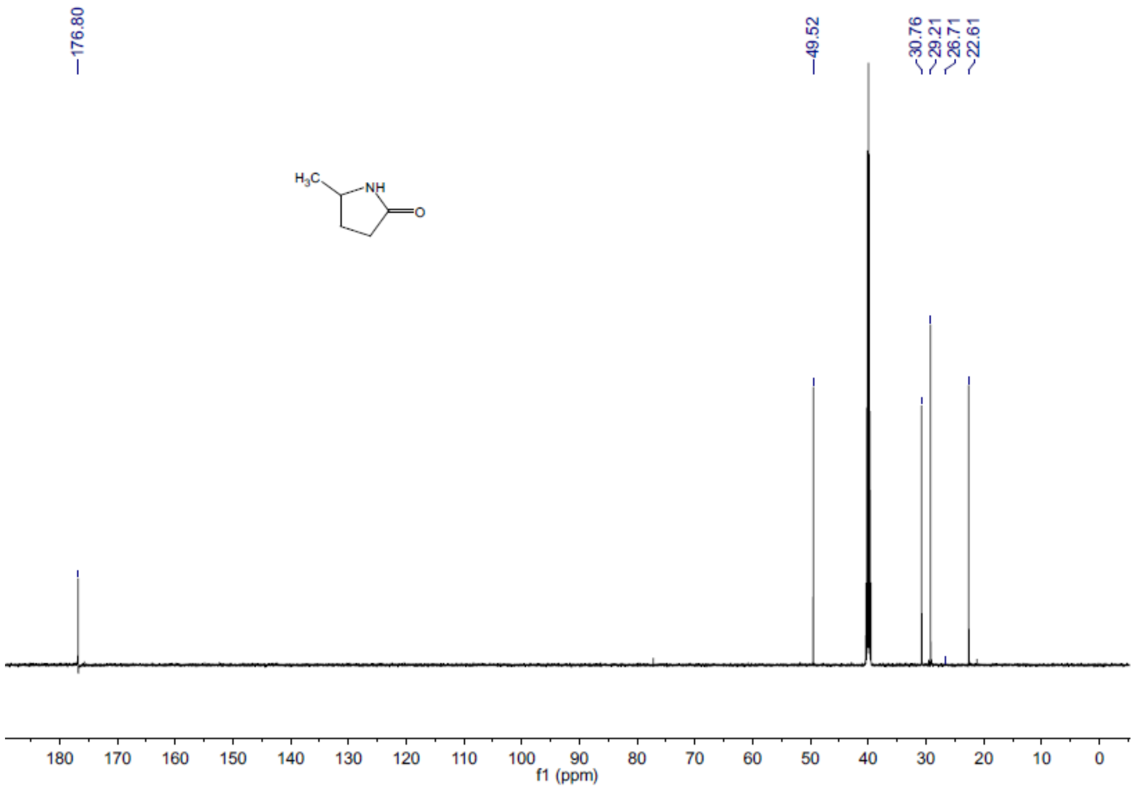

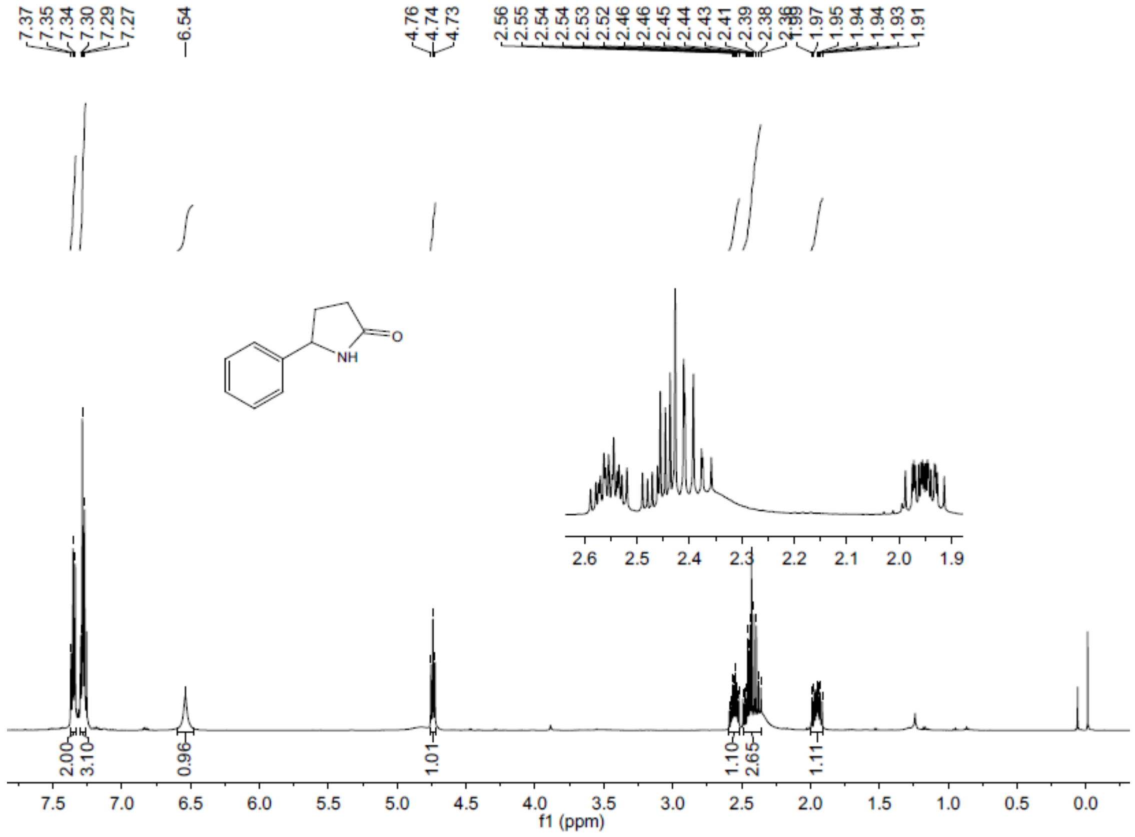
7.37
7.35
7.34
7.30
7.29
7.27
6.54
4.76
4.74
4.73
2.56
2.55
2.54
2.54
2.53
2.52
2.46
2.45
2.44
2.43
2.41
2.39
2.38
2.36
1.99
1.97
1.95
1.94
1.94
1.93
1.91
NH
O
2.00
3.10
0.96
1.01
1.10
2.65
1.11
f1 (ppm)

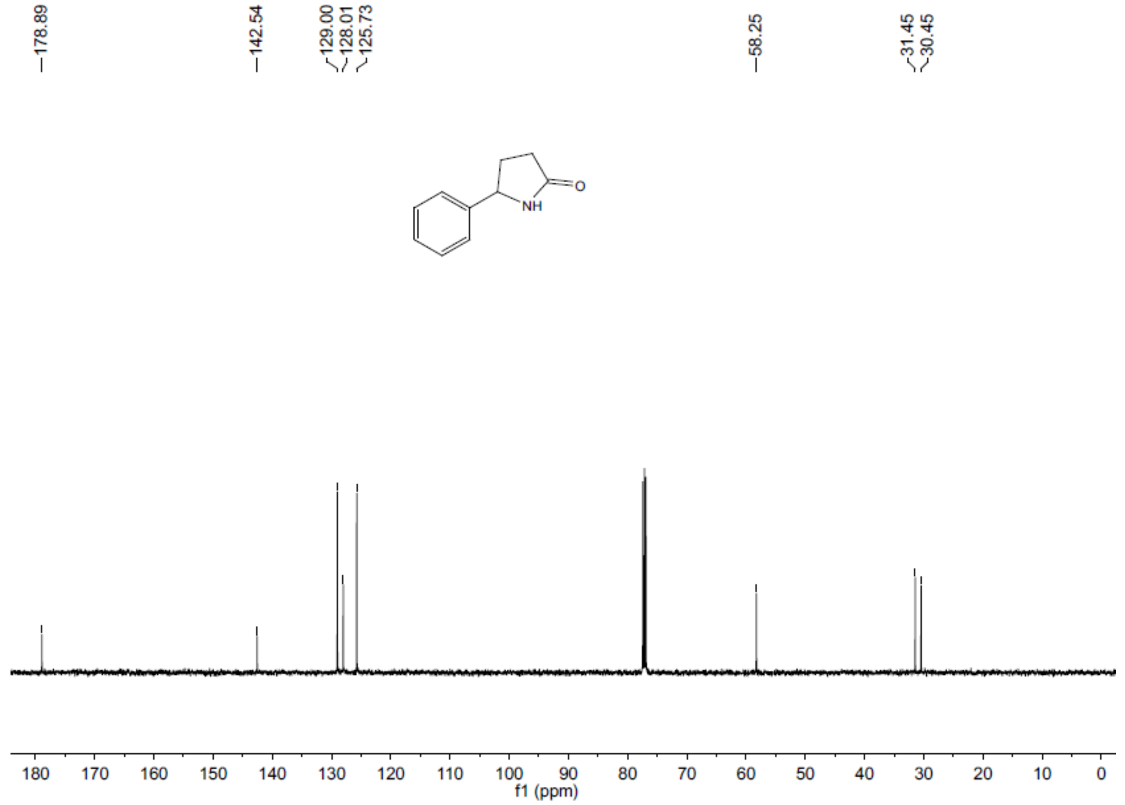
178.89
142.54
129.00
128.01
125.73
58.25
31.45
30.45
NH
O
f1 (ppm)

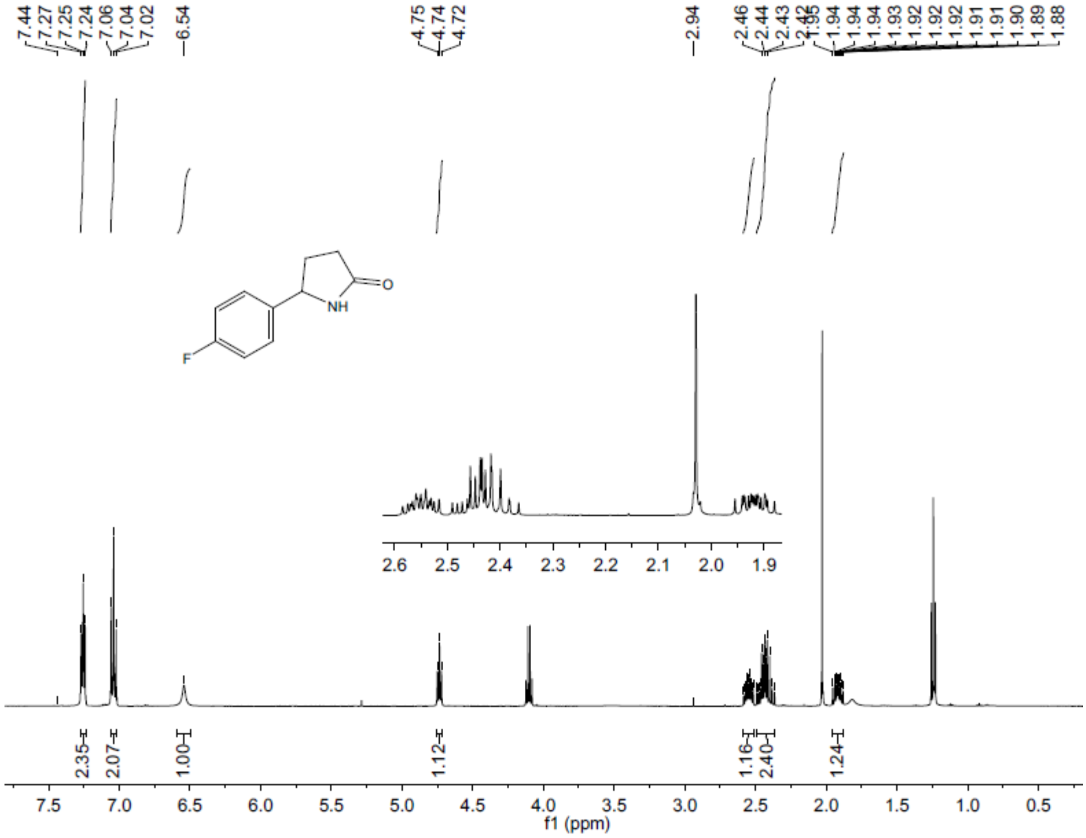
7.44
7.27
7.25
7.24
7.06
7.04
7.02
6.54
4.75
4.74
4.72
2.94
2.46
2.44
2.43
2.42
1.95
1.94
1.94
1.94
1.93
1.92
1.92
1.92
1.91
1.91
1.90
1.89
1.88
NH
O
F
2.35
2.07
1.00
1.12
1.16
2.40
1.24
f1 (ppm)

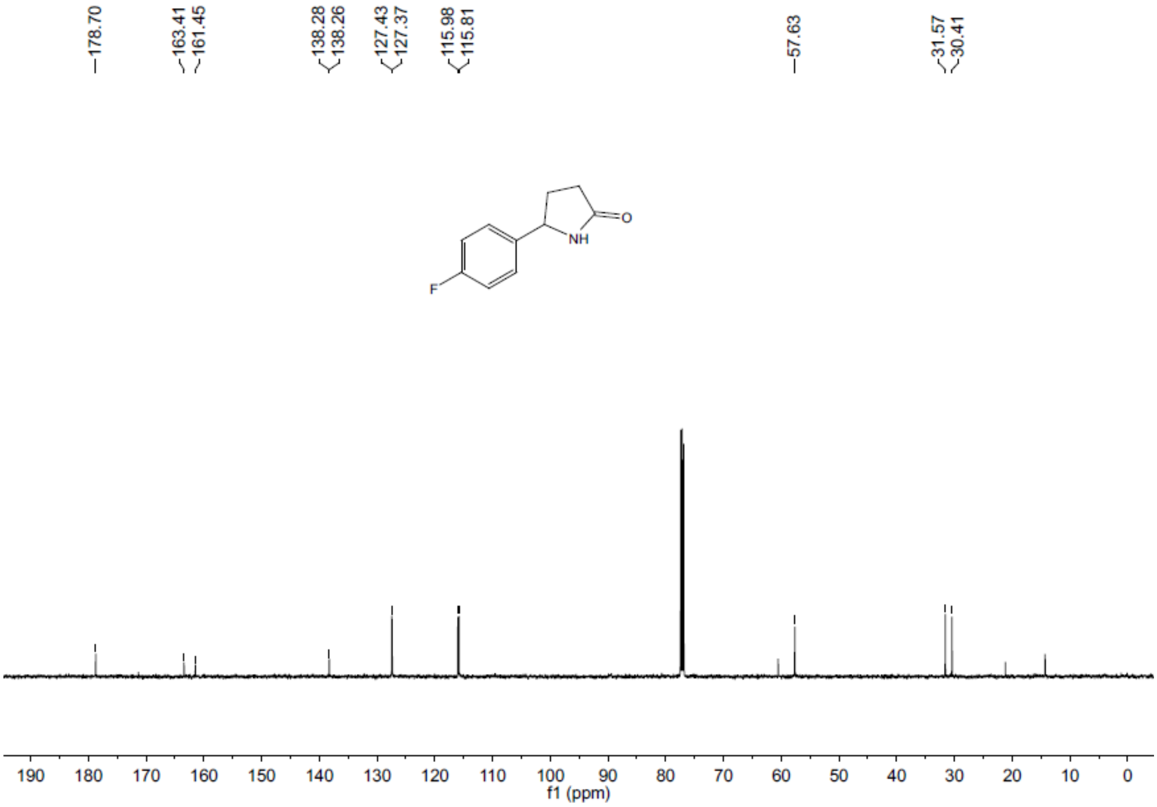
178.70
163.41
161.45
138.28
138.26
127.43
127.37
115.98
115.81
57.63
31.57
30.41
NH
O
F
f1 (ppm)

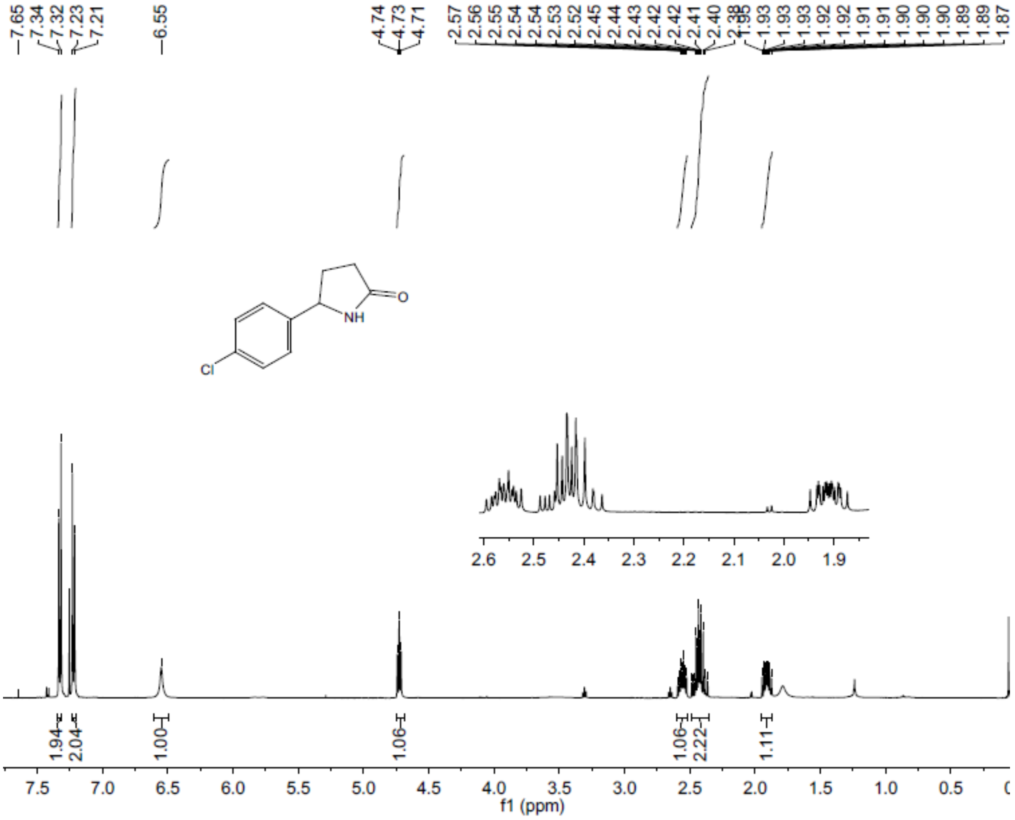
7.65 7.34 7.32 7.23 7.21
6.55
4.74 4.73 4.71
2.57 2.56 2.55 2.54 2.54 2.53 2.52 2.45 2.44 2.43 2.42 2.42 2.41 2.40 2.38 1.95 1.93 1.93 1.93 1.92 1.92 1.91 1.91 1.90 1.90 1.90 1.89 1.89 1.87
NH
O
Cl
1.94 2.04 1.00 1.06 1.06 2.22 1.11
f1 (ppm)

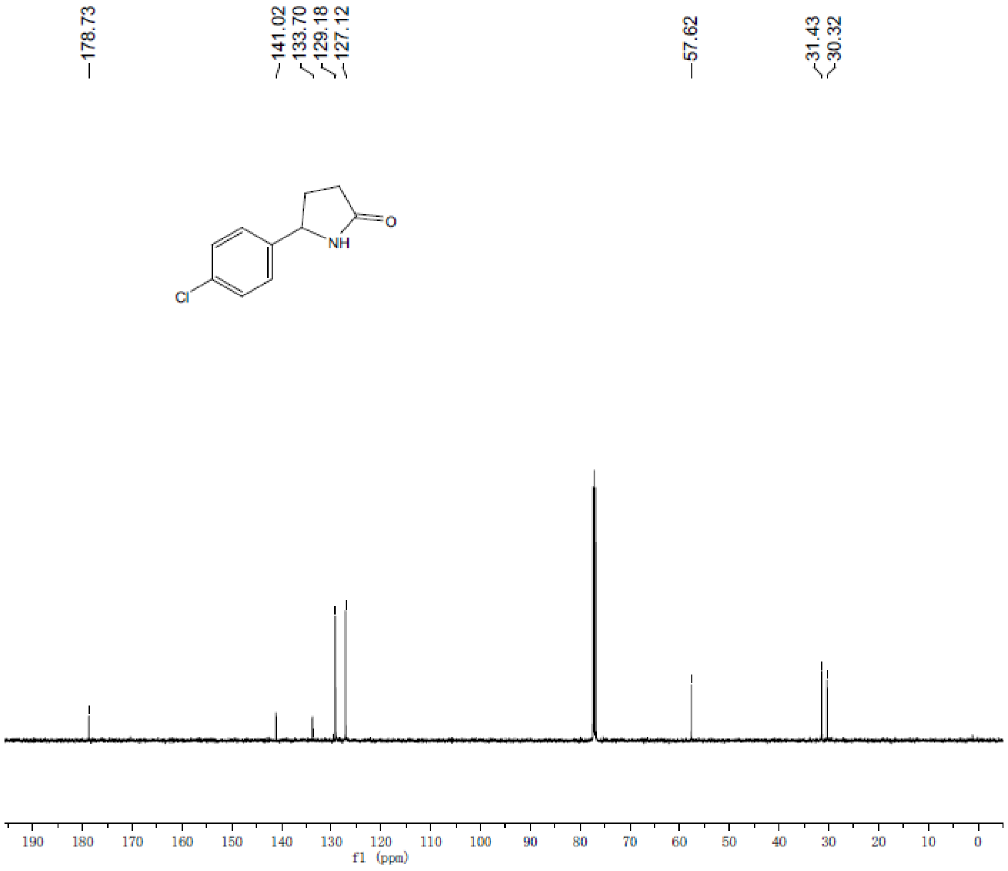
178.73
141.02 133.70 129.18 127.12
57.62
31.43 30.32
NH
O
Cl
f1 (ppm)

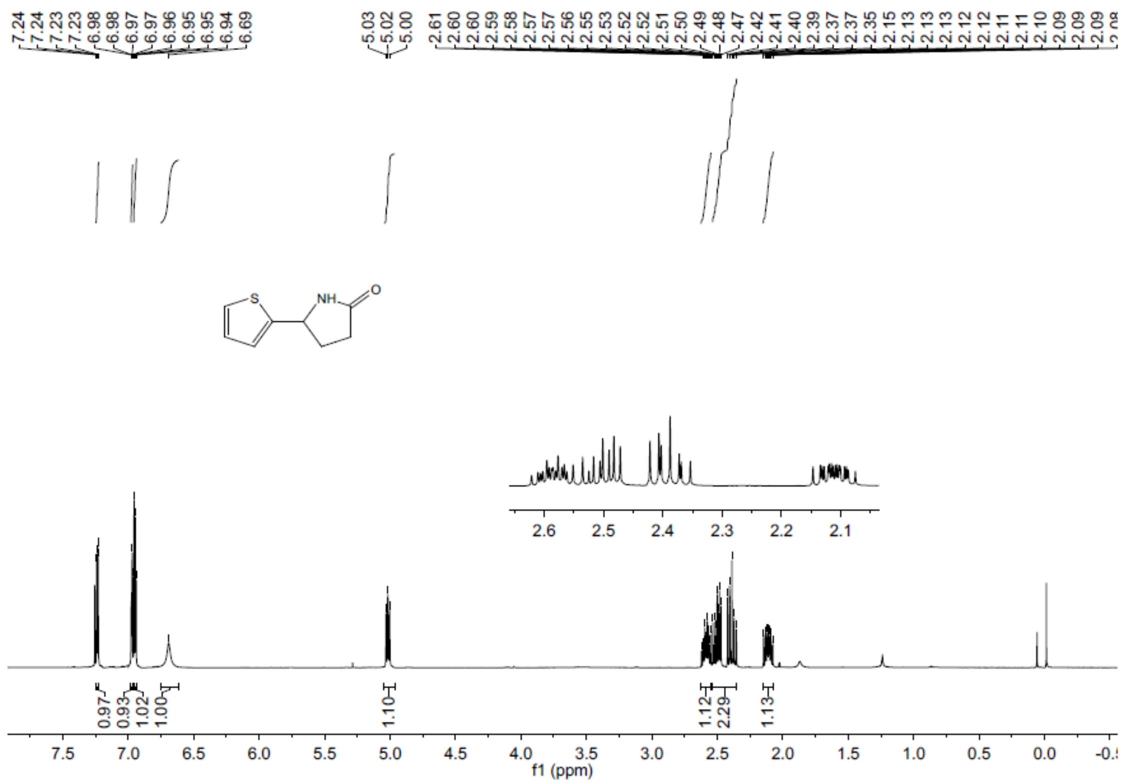

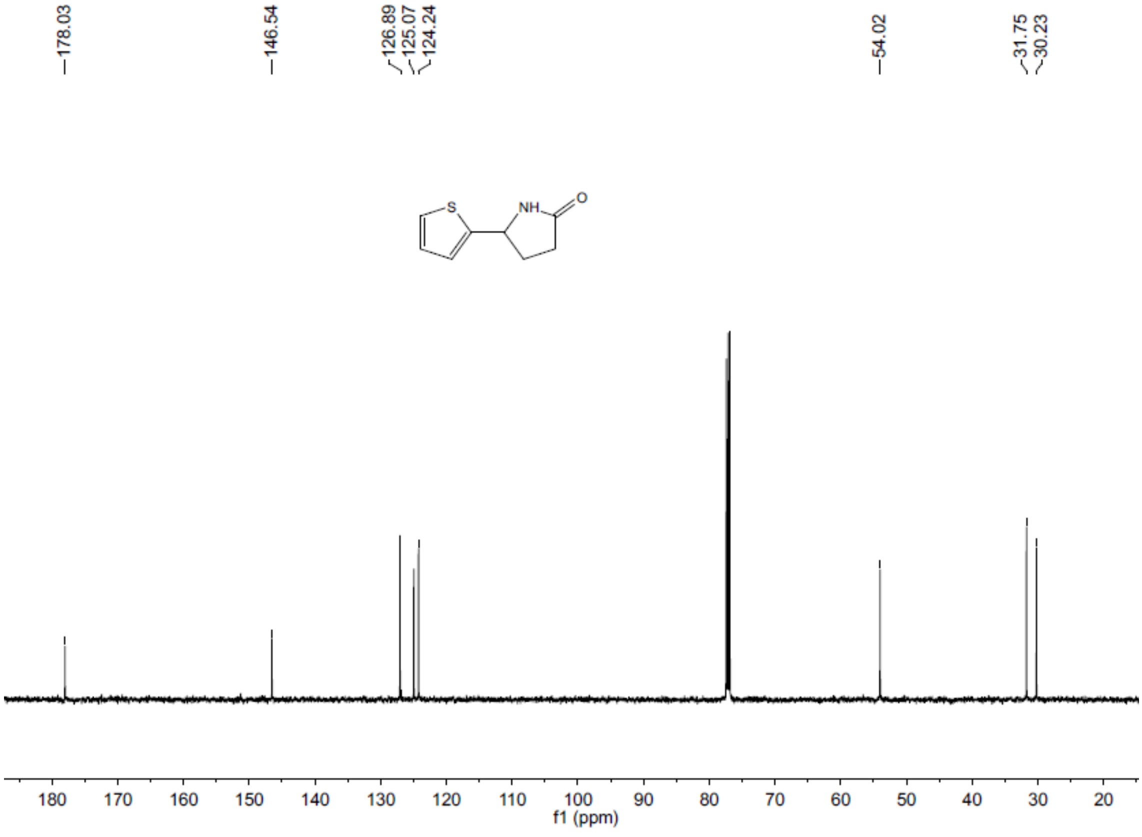

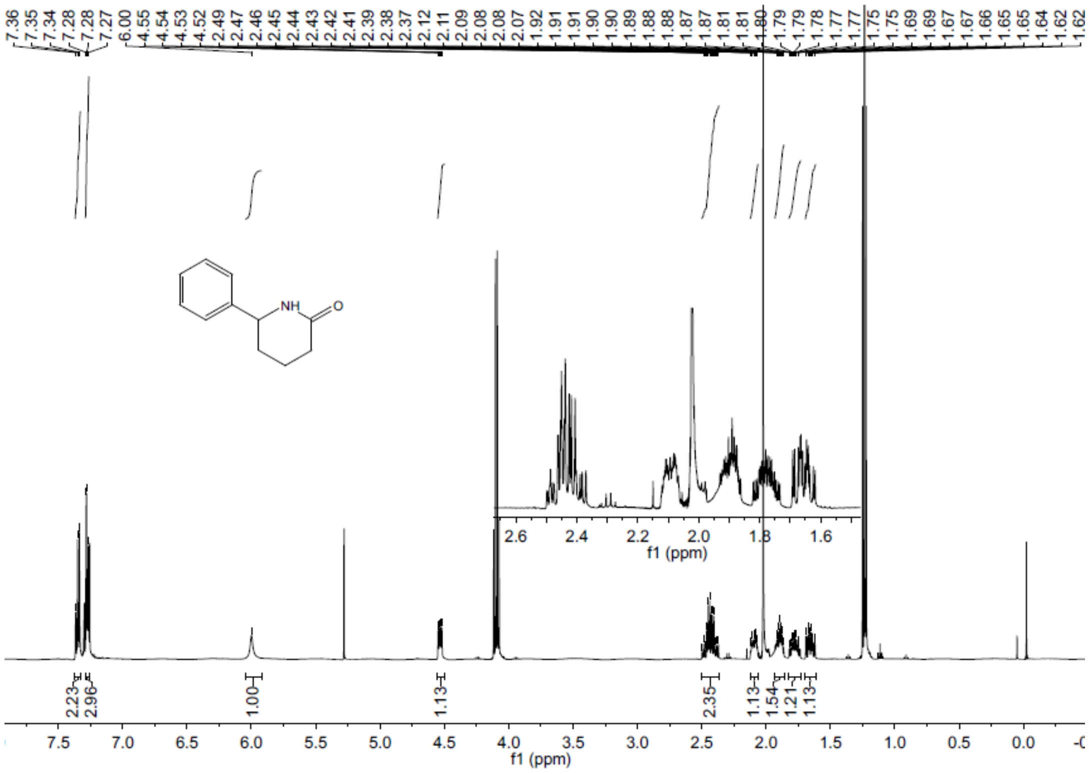

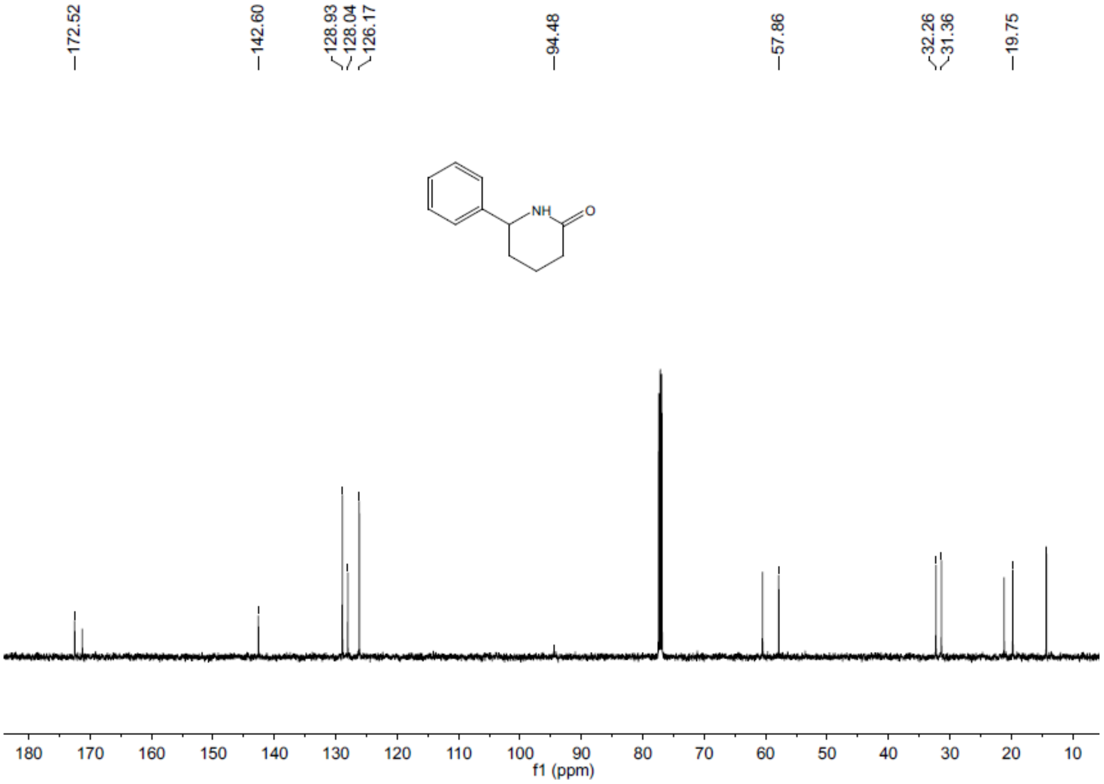

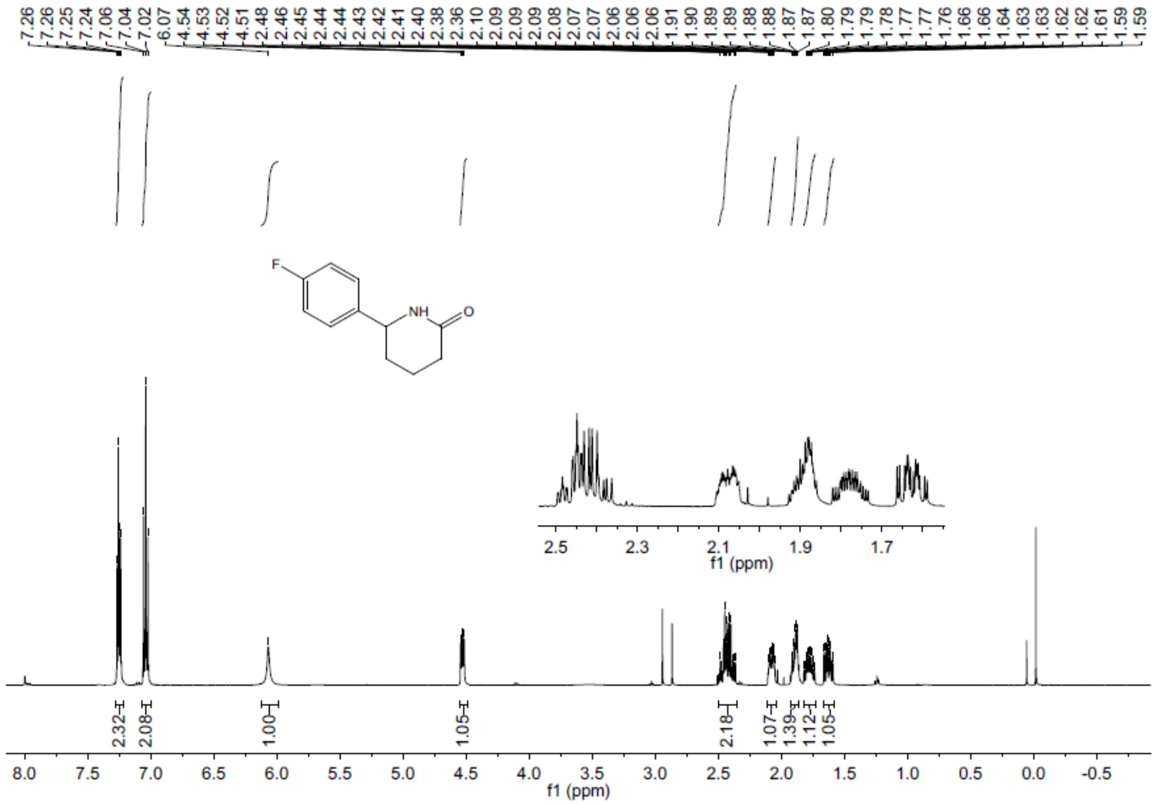
7.26 7.26 7.25 7.24 7.06 7.04 7.02 6.07 4.54 4.53 4.52 4.51 2.48 2.46 2.45 2.44 2.44 2.43 2.42 2.41 2.40 2.38 2.36 2.10 2.09 2.09 2.09 2.08 2.07 2.07 2.06 2.06 2.06 1.91 1.90 1.89 1.89 1.88 1.88 1.87 1.87 1.80 1.79 1.78 1.77 1.77 1.76 1.66 1.66 1.64 1.63 1.63 1.62 1.62 1.61 1.59 1.59
F
NH
O
2.5 2.3 2.1 1.9 1.7
f1 (ppm)
2.32 2.08 1.00 1.05 2.18 1.07 1.39 1.12 1.05
8.0 7.5 7.0 6.5 6.0 5.5 5.0 4.5 4.0 3.5 3.0 2.5 2.0 1.5 1.0 0.5 0.0 -0.5
f1 (ppm)

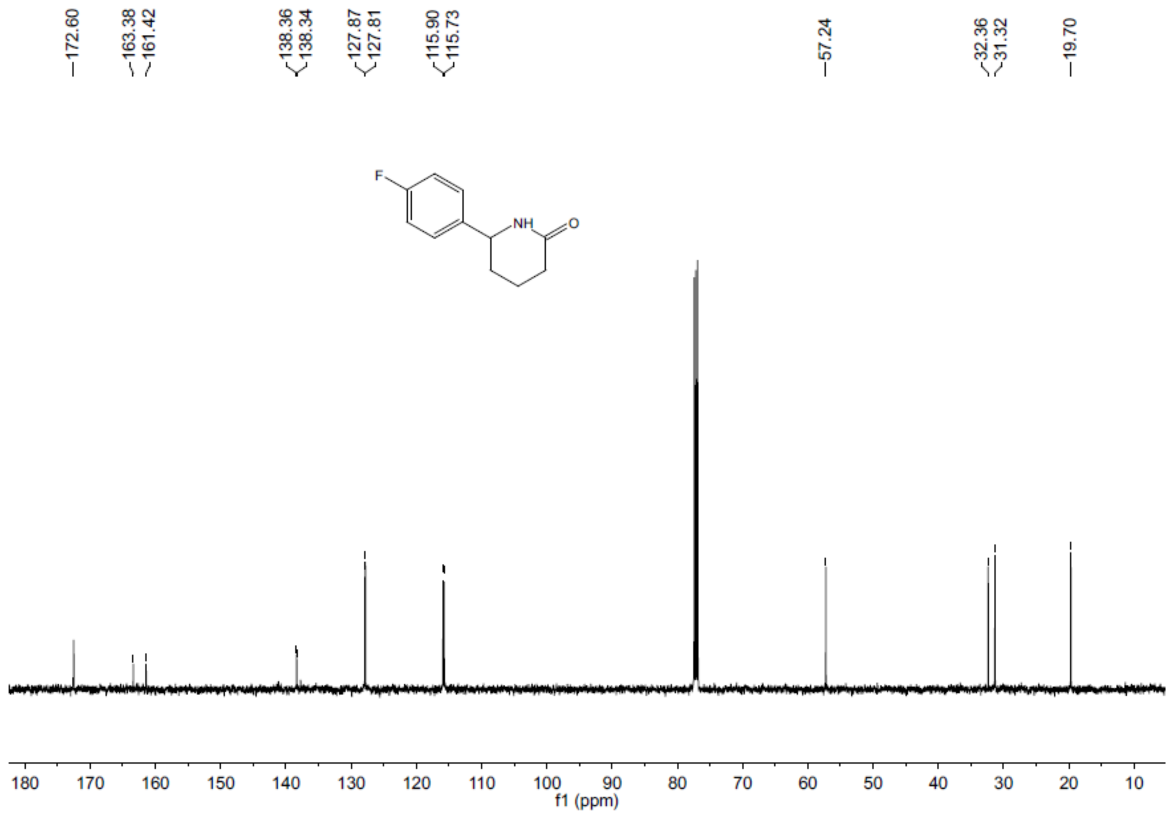
172.60 163.38 161.42 138.36 138.34 127.87 127.81 115.90 115.73 57.24 32.36 31.32 19.70
F
NH
O
180 170 160 150 140 130 120 110 100 90 80 70 60 50 40 30 20 10
f1 (ppm)

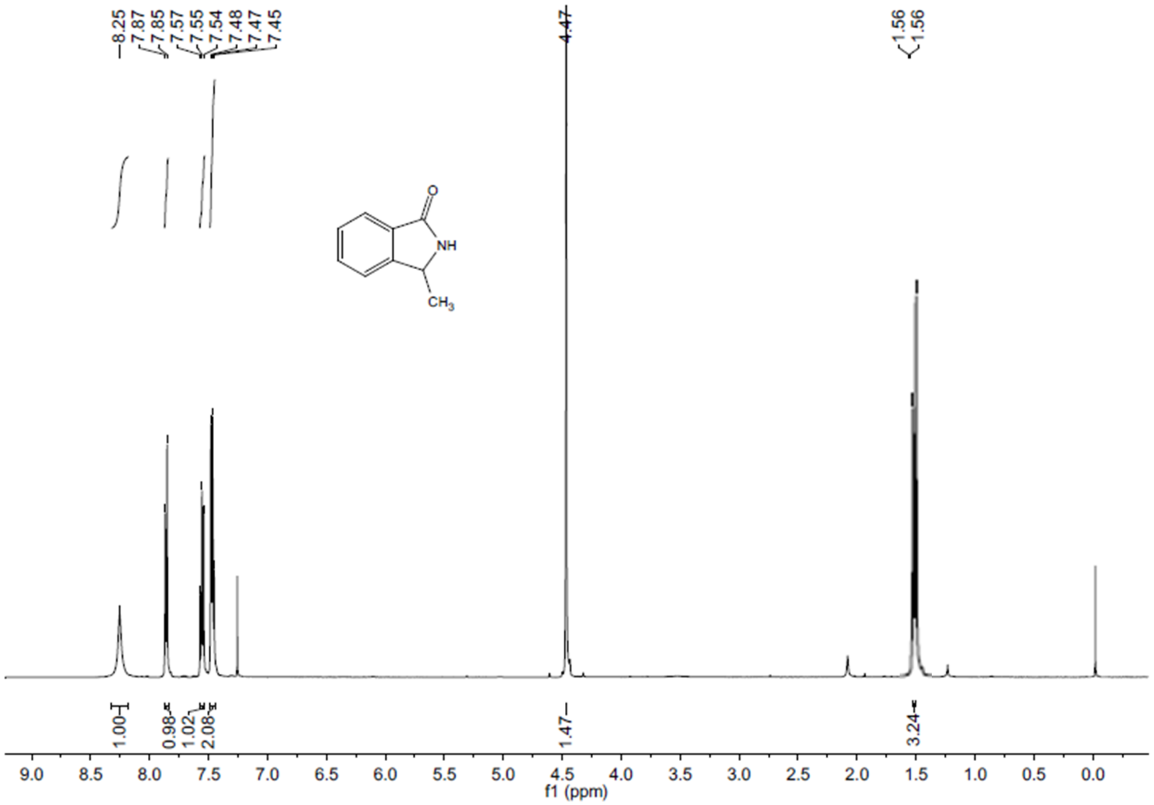

8.25
7.87
7.85
7.57
7.55
7.54
7.48
7.47
7.45
4.47
1.56
1.56
NH
CH3
1.00
0.98
1.02
2.08
1.47
3.24
f1 (ppm)

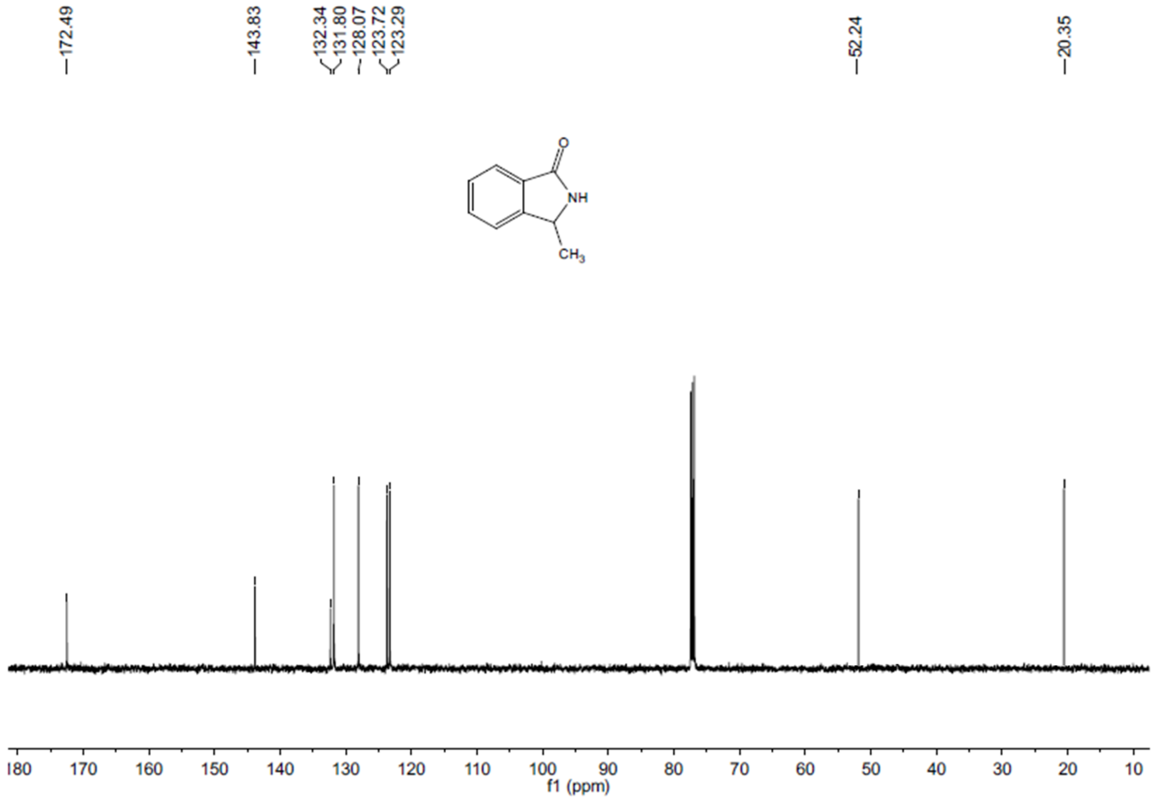

172.49
143.83
132.34
131.80
128.07
123.72
123.29
52.24
20.35
NH
CH3
f1 (ppm)

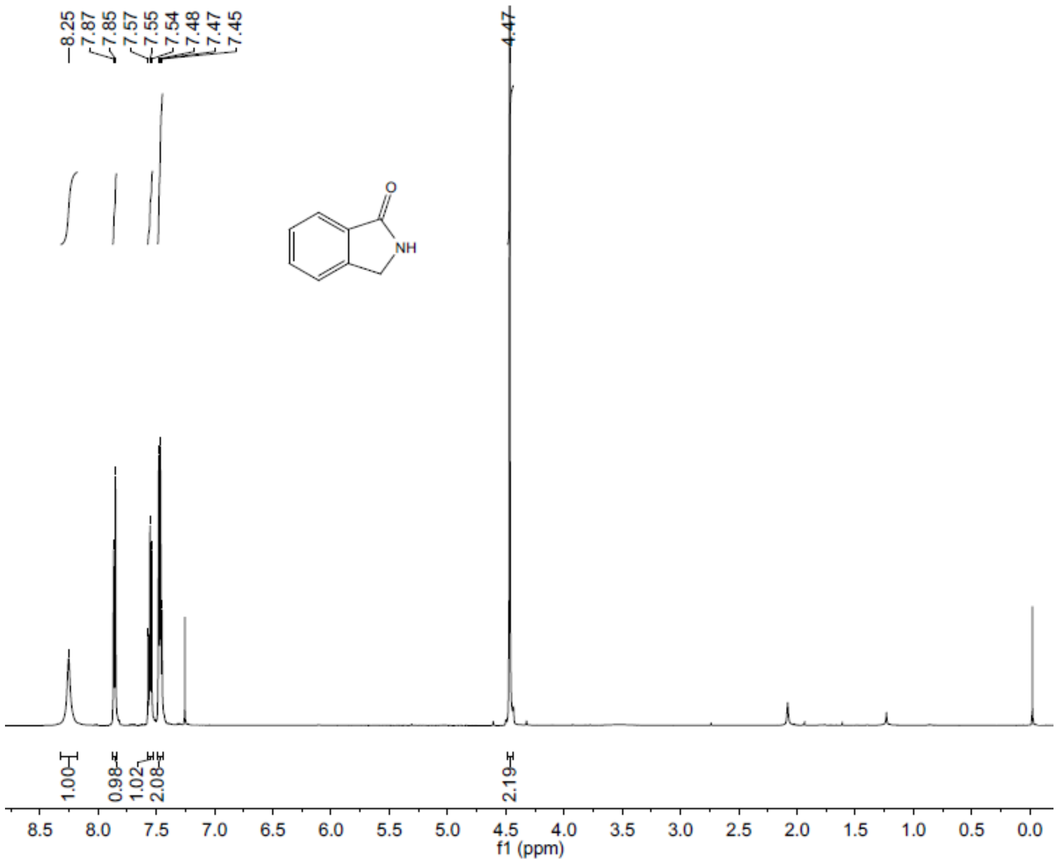
8.25
7.87
7.85
7.57
7.55
7.54
7.48
7.47
7.45
4.47
O
NH
1.00
0.98
1.02
2.08
2.19
8.5 8.0 7.5 7.0 6.5 6.0 5.5 5.0 4.5 4.0 3.5 3.0 2.5 2.0 1.5 1.0 0.5 0.0
f1 (ppm)

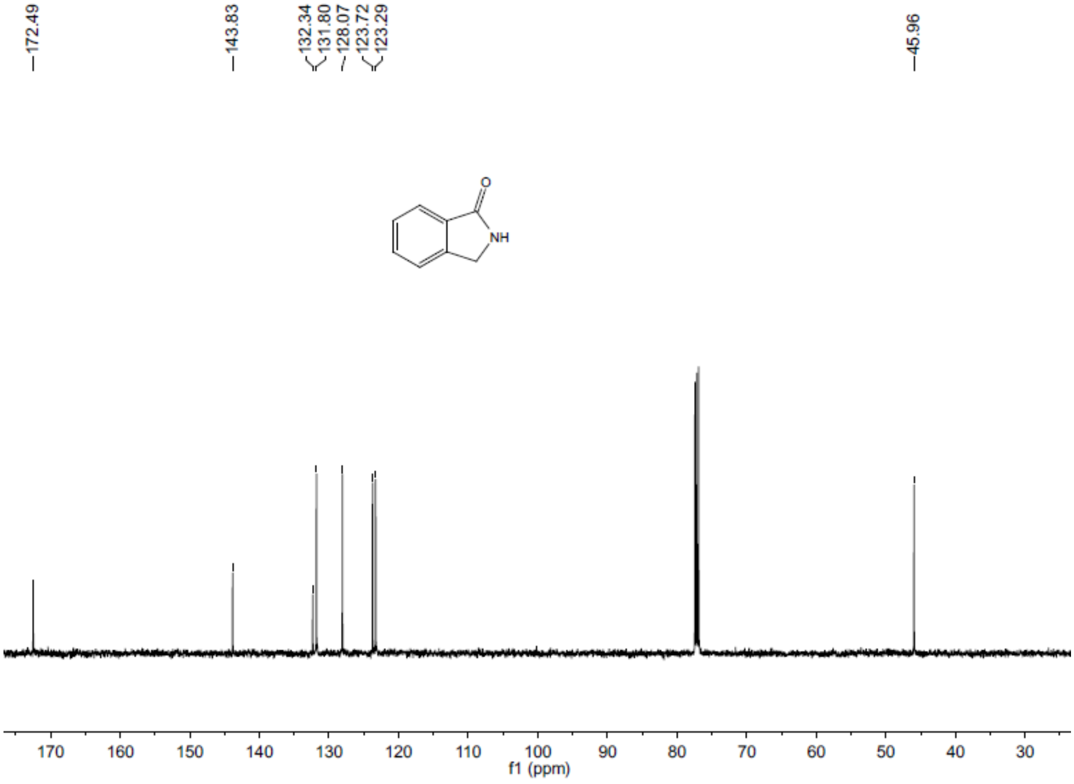
172.49
143.83
132.34
131.80
128.07
123.72
123.29
45.96
O
NH
170 160 150 140 130 120 110 100 90 80 70 60 50 40 30
f1 (ppm)

Computational Information:

Optimized geometries of some reaction intermediates and transition states:

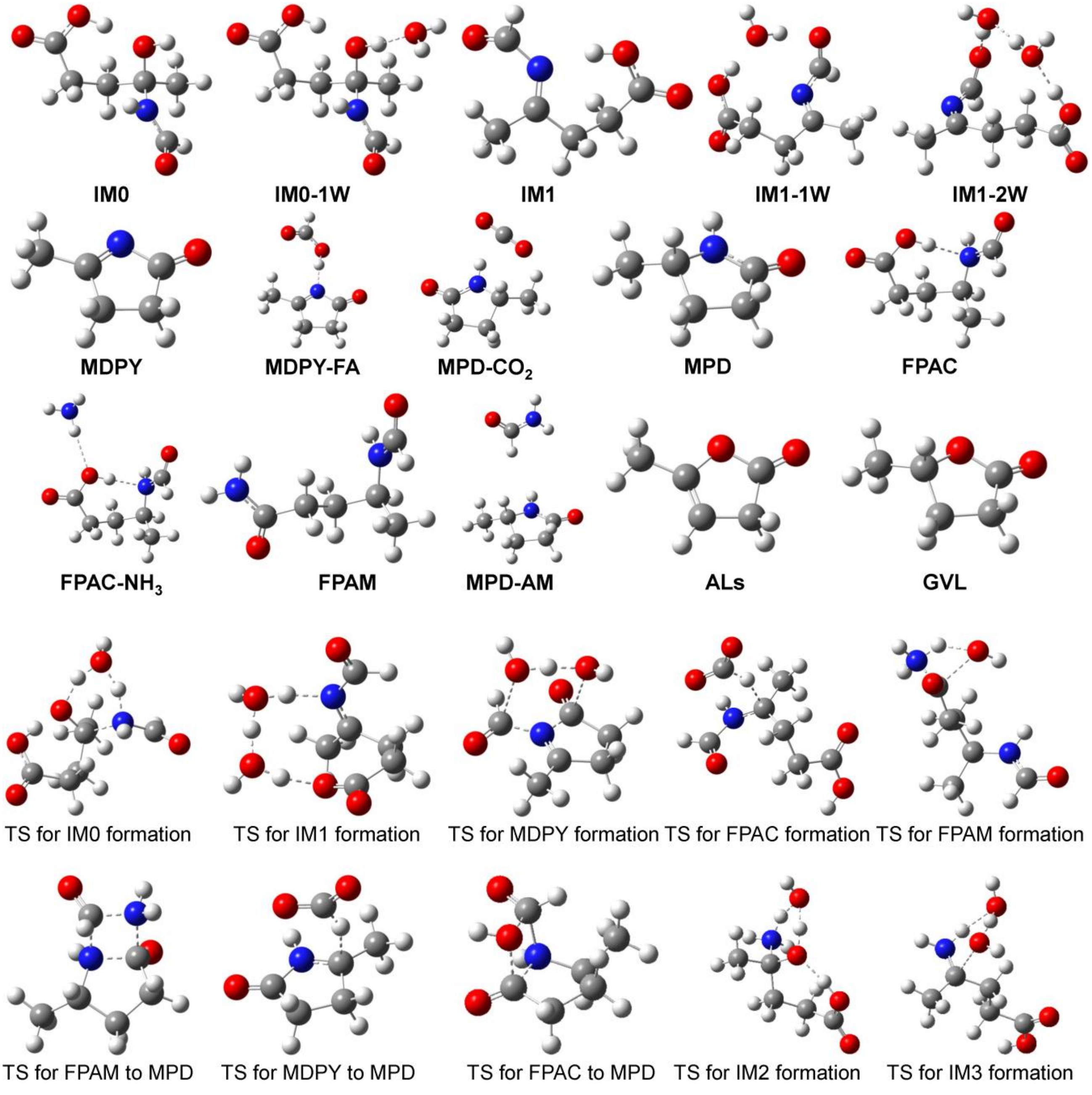

Structural coordinates of some important intermediates and transition states:

IM0-1W:

C	-0.53273600	0.05052200	-0.66685600
C	-1.39431100	-0.19902500	-1.90597500
H	-1.66647400	0.72965000	-2.40973400
H	-0.81577700	-0.80580200	-2.60323700
H	-2.30502500	-0.74221300	-1.64646400
O	-0.09467800	-1.19068600	-0.11668800
C	0.73714500	0.83951600	-1.05100600
H	1.22542400	0.31205300	-1.87499800
H	0.39961100	1.79712500	-1.45191000
C	1.76025300	1.13342200	0.06843200
H	2.29047400	2.05519300	-0.16885100
H	1.25767000	1.31653300	1.02472600
C	2.84277900	0.09179200	0.32496000
O	4.00733800	0.39705100	0.49012300
O	2.47487400	-1.19055300	0.39313700
H	1.49789900	-1.29241800	0.25031900
C	-2.42809900	1.45587000	0.29671500
H	-2.85302500	1.49854300	-0.71365700
O	-2.96760300	2.01777500	1.24571200
N	-1.28489100	0.74226300	0.39465300
H	-0.87799100	0.69818400	1.32206000
H	-2.69461800	-2.63840400	1.33734500
O	-2.04302600	-2.92883000	0.68785200
H	-0.84873700	-1.76793900	0.14477600
H	-2.54062500	-3.42699000	0.02834100

IM1:

C	-0.90651600	0.77566600	-0.15146100
C	-2.06296700	1.67078600	-0.49210900
H	-2.35492800	2.23015400	0.40240000
H	-1.75223200	2.40508600	-1.23926200
H	-2.92830500	1.12109700	-0.85680800
C	0.41839400	1.46738700	0.05935500
H	0.77668300	1.79747900	-0.92384100
H	0.21408500	2.39157900	0.60778300
C	1.51584700	0.69428000	0.81675700
H	2.24857700	1.40861200	1.18910900
H	1.07608600	0.19249400	1.68510400
C	2.29314500	-0.34160500	0.01673600
O	3.49624200	-0.29517600	-0.13422400

O	1.58897000	-1.35127900	-0.50938800
H	0.62296300	-1.22487500	-0.31418200
N	-0.97164600	-0.50126800	-0.05676600
C	-2.13503700	-1.26477900	-0.13420100
H	-2.10317600	-2.01204700	-0.94251100
O	-3.05214000	-1.20493300	0.65600100

IM1-2W:

C	-1.25282200	-1.33219300	0.60892000
C	-1.83051200	-2.04114000	1.80244000
H	-1.45874900	-3.07019800	1.83104900
H	-1.50215600	-1.55690100	2.72620800
H	-2.91801900	-2.05319200	1.75941700
O	1.05296500	2.30665800	1.15998300
C	0.24225700	-1.08823600	0.66340200
H	0.39873300	-0.34261700	1.45156000
H	0.70540600	-2.00868600	1.03311300
C	0.91847600	-0.64953600	-0.64358900
H	0.76366900	-1.39665400	-1.42256200
H	0.51035700	0.30166200	-0.99158300
C	2.42084400	-0.49551800	-0.46085100
O	3.22445900	-1.29928700	-0.88690800
O	2.83840000	0.57674900	0.21591600
H	2.10496500	1.18694900	0.50430400
C	-1.96850600	-0.34303500	-1.51920600
H	-1.96569200	-1.01416500	-2.39096200
O	-2.06612800	0.87609700	-1.66549100
N	-2.02511800	-0.99030300	-0.33773400
H	-1.76612900	2.10301500	-0.40023900
H	-2.15175800	2.83946500	0.91271200
O	-1.46623400	2.78736100	0.23752400
H	1.46591300	3.16110000	1.32763300
H	0.14317200	2.49967500	0.82860300

MDPY:

C	-0.98488700	-0.15551500	0.00001300
C	-2.41606900	-0.55979800	-0.00001100
H	-2.91951700	-0.14077300	0.87713700
H	-2.91937500	-0.14104700	-0.87737900
H	-2.52253900	-1.64345800	0.00013200
C	-0.56661400	1.29895900	0.00004100
H	-0.97612500	1.80686200	-0.87757500
H	-0.97602300	1.80677600	0.87775600
C	0.95884000	1.20301100	-0.00004100

H	1.42579700	1.64902000	-0.88027500
H	1.42591500	1.64910300	0.88008500
C	1.21382200	-0.30158400	-0.00000500
O	2.29444000	-0.85055100	0.00001800
N	-0.01774300	-1.01321600	0.00000000

MDPY-FA (HCOOH):

C	-0.84441500	1.03076500	-0.09049500
C	-0.02731500	2.26162500	-0.23856700
H	-0.27041200	2.73676000	-1.19550000
H	-0.29223400	2.98125800	0.54124000
H	1.03826500	2.04301400	-0.19779100
C	-2.34519300	1.07350900	0.06484400
H	-2.60721700	1.64615900	0.95880500
H	-2.79252400	1.59554200	-0.78534500
C	-2.72240700	-0.40559900	0.15170300
H	-3.20822400	-0.67967200	1.08978500
H	-3.36858000	-0.73961800	-0.66228000
C	-1.38126800	-1.11906300	0.05125400
O	-1.17832700	-2.31038100	0.08214500
N	-0.33373100	-0.15915300	-0.09372200
C	3.20452400	-0.37369300	0.00994300
H	4.16776800	-0.86357700	-0.18820700
O	3.10916100	0.69485400	0.57710000
O	2.20230600	-1.10164100	-0.44937400
H	1.30060200	-0.67371600	-0.27571700

MPD-CO_2:

C	1.19145600	0.99267900	-0.17989200
C	1.43606100	2.35003200	-0.82709700
H	0.97010200	3.13664500	-0.22753400
H	2.50742100	2.55680300	-0.89863000
H	1.00452800	2.39512000	-1.83073500
C	1.80972100	0.82870500	1.22779600
H	2.71257500	1.44232200	1.29514600
H	1.12322800	1.15282400	2.01018200
C	2.17953200	-0.66024600	1.31570900
H	3.08342000	-0.86716100	1.88937400
H	1.37111400	-1.26199400	1.74471900
C	2.35003500	-1.08946800	-0.13699300
O	2.84674800	-2.14065600	-0.53911200
N	1.83416600	-0.10734100	-0.91316600
C	-4.72810100	-0.26259300	0.01683000
H	0.11068900	0.80727400	-0.13542100

O	-4.83861900	0.79637200	0.47887100
O	-4.61755600	-1.32152100	-0.44523900
H	1.72095400	-0.23866200	-1.90921800

FPAC-NH_3:

C	-1.91012300	-0.39351700	0.62701300
C	-2.78875500	-0.77236900	-0.56465600
H	-3.19525600	-1.77108900	-0.39296100
H	-3.63324700	-0.08973400	-0.67380400
H	-2.23883900	-0.79016500	-1.50848300
C	-0.90014800	-1.49432900	1.01307200
H	-0.30689000	-1.15356200	1.86773600
H	-1.49764700	-2.32998700	1.38562400
C	0.02819900	-2.06523400	-0.08509300
H	0.28019500	-3.09579400	0.16336900
H	-0.49123200	-2.10568800	-1.04875900
C	1.35715800	-1.37475400	-0.33781700
O	2.39219300	-1.98007400	-0.51079200
O	1.36705700	-0.03004000	-0.40699000
H	0.47715600	0.34780600	-0.23754500
N	-1.20844000	0.90901600	0.43975200
C	-1.61025200	1.92205900	-0.39156100
H	-2.25286800	1.60156400	-1.21996300
O	-1.24644800	3.08120000	-0.26534700
H	-0.76699800	1.26079300	1.28638900
H	-2.56499400	-0.26945600	1.49556500
N	3.94216900	1.86864500	0.48292300
H	4.82317200	1.44167400	0.21098700
H	3.87340100	2.74539600	-0.02604000
H	3.19904700	1.26477900	0.13844100

FPAM:

C	1.07377300	1.06999800	0.53910600
C	1.38316300	1.89609900	-0.70913000
H	0.79116400	2.81293800	-0.67236700
H	2.43643000	2.18210600	-0.74796500
H	1.13419200	1.37470500	-1.63572000
C	-0.42935900	0.77770300	0.72048800
H	-0.56634200	0.25211700	1.67207500
H	-0.94866700	1.73460600	0.81635900
C	-1.07154900	-0.04266800	-0.40871000
H	-1.00531400	0.49938800	-1.35661100
H	-0.54690900	-0.99184900	-0.53727900
C	-2.55259200	-0.27557800	-0.14988200

O	-3.33891600	0.65948400	0.00689100
N	1.84630500	-0.18625100	0.60908800
C	2.72181800	-0.69286100	-0.27584400
H	2.85652000	-0.08590700	-1.17960300
O	3.33689900	-1.74925800	-0.11636600
H	1.72486900	-0.73548900	1.45292600
H	1.37843200	1.66475000	1.40723600
N	-2.95366100	-1.56703100	-0.10439200
H	-3.92725900	-1.77996400	0.05668000
H	-2.31101200	-2.33237900	-0.22896800

MPD-AM (H_2NCHO):

C	-1.27287900	1.08205700	-0.11412000
C	-1.65630100	2.33981100	-0.88345600
H	-2.71047100	2.31008200	-1.17265500
H	-1.49256600	3.22194400	-0.25870700
H	-1.05009500	2.45253600	-1.78644600
C	-2.11471400	0.81938700	1.15563300
H	-1.68250500	1.30108100	2.03293100
H	-3.11993600	1.22464700	1.00877800
C	-2.17809900	-0.71201700	1.26633400
H	-1.36647000	-1.11602300	1.88091200
H	-3.11642900	-1.09618500	1.66749400
C	-1.96021600	-1.19930300	-0.16162100
O	-2.13832700	-2.34114200	-0.58346500
N	-1.51189800	-0.14653400	-0.88523600
H	-1.17911500	-0.27038100	-1.83185000
H	-0.20888600	1.13487800	0.14988700
C	3.79632400	0.05953500	0.24557400
H	2.77115300	0.22372200	0.61367900
O	4.67211400	0.90966600	0.36154300
N	3.96119200	-1.14679900	-0.32731300
H	4.85916500	-1.41728300	-0.70327000
H	3.19610900	-1.80070500	-0.38758400

GLs:

C	1.20264600	-0.25696800	0.00000000
O	-0.00076100	-0.91463400	-0.00005400
C	-1.04221000	0.04295000	-0.00009400
C	-0.56806100	1.28707900	-0.00007100
C	0.93054100	1.23550100	0.00008200
H	-1.16719800	2.18434700	-0.00037900
H	1.39703600	1.69185300	-0.87887500
H	1.39662800	1.69157600	0.87940100

C	-2.40780400	-0.53324200	0.00004900
H	-2.56431300	-1.15946800	0.88333200
H	-3.14905500	0.26613700	0.00024700
H	-2.56478400	-1.15963200	-0.88300400
O	2.24588800	-0.85620700	-0.00001100

GVL:

C	1.21788400	-0.27149100	0.01074500
O	0.06708300	-0.95255900	-0.16687500
C	-1.06150300	-0.03562200	-0.40457400
C	-0.57062600	1.31049500	0.14534100
C	0.94639300	1.21937400	-0.03768200
H	-0.82336400	1.39271300	1.20591500
H	1.26624600	1.58167100	-1.02041400
H	1.53557300	1.74015800	0.71614300
C	-2.30395600	-0.60794000	0.24139800
H	-2.16616900	-0.71087200	1.32064900
H	-3.14716100	0.06445500	0.06247700
H	-2.55208500	-1.58439000	-0.17969200
O	2.27335100	-0.83926400	0.17133900
H	-1.02512900	2.14937400	-0.38138100
H	-1.18053100	0.01258000	-1.49078700

TS-1 for IM0 formation:

C	-0.47092200	0.27869100	0.68057800
C	-1.27776700	0.56829500	1.94909900
H	-1.71378900	-0.32909700	2.39357600
H	-0.58913800	1.00505200	2.67408800
H	-2.06941500	1.29672500	1.76026000
O	0.12441000	1.39870600	0.17951700
C	0.52556300	-0.87639200	0.90821600
H	1.06855900	-0.64119800	1.82848400
H	-0.03332800	-1.79234100	1.11607000
C	1.53694300	-1.16326100	-0.22263400
H	1.89826100	-2.18663500	-0.12699800
H	1.06753600	-1.09932800	-1.21146900
C	2.77374800	-0.26897700	-0.27070300
O	3.89240100	-0.72928200	-0.42857400
O	2.57902800	1.03515600	-0.15793300
H	1.57264700	1.24376600	-0.04083100
C	-2.62923300	-1.03886100	-0.14871900
H	-3.21702500	-0.69548500	0.70991800
O	-2.91595200	-2.00796700	-0.80359700
N	-1.51769300	-0.20582000	-0.46210900

H	-0.99906400	-0.64229500	-1.22678500
H	-1.85859700	0.92868700	-0.92478200
O	-1.68891800	2.12197700	-1.24084200
H	-0.82145300	2.09327800	-0.60620400
H	-2.35907600	2.71391000	-0.87414100

TS-2 for IM1 formation:

C	-0.20077400	-0.95579800	1.03904900
C	-0.55958600	-0.41098600	2.38081200
H	-1.54335000	0.05444700	2.39398600
H	-0.56714500	-1.25522200	3.08244400
H	0.20043400	0.29019100	2.72417100
O	-0.79902700	2.75566400	-0.23084000
C	1.18916500	-1.53171100	0.88244300
H	1.71881800	-1.38332600	1.82385100
H	1.10681900	-2.61615100	0.74083200
C	2.00717500	-0.92422000	-0.27262100
H	3.05024300	-1.23380500	-0.17785000
H	1.67454200	-1.30762900	-1.24223000
C	1.94654400	0.61190900	-0.35777000
O	2.71036200	1.18914000	-1.14853800
O	1.07040400	1.16681100	0.39198500
H	0.04745400	2.22866700	0.00905400
C	-0.98205400	-1.74363500	-1.04650300
H	-0.30575700	-2.61040800	-0.98340000
O	-1.62461600	-1.51709000	-2.04949100
N	-1.12735100	-0.98965900	0.11886500
H	-2.09883200	0.17502500	0.01040800
H	-3.28893500	1.26199500	0.47606400
O	-2.58544700	1.10739900	-0.16902100
H	-0.86324600	3.51409900	0.36216300
H	-1.81583000	1.92097000	-0.15676700

TS-3 for MDPY formation from IM1:

C	0.12140200	1.30551700	0.17552000
C	1.24344300	2.13151900	0.65999400
H	0.88115500	2.88002700	1.36222200
H	2.02439700	1.51579200	1.10742400
H	1.70002900	2.63963500	-0.19714700
C	-1.30963800	1.65270800	0.40882600
H	-1.48753800	1.56172600	1.48707500
H	-1.48411000	2.70007300	0.15105600
C	-2.11650500	0.64687900	-0.41056300
H	-2.94500700	0.19494600	0.12583500

H	-2.49682700	1.09746000	-1.33295200
C	-1.11641500	-0.40711400	-0.80403200
O	-1.21229800	-1.39839100	-1.46082500
O	-1.16754100	-1.40128000	1.42816700
H	-1.39290600	-0.95288600	2.25065700
N	0.22773300	0.18338300	-0.48595700
C	1.44878800	-0.50315200	-0.89263200
H	1.21740500	-1.48049400	-1.31348500
O	2.51278100	0.04638300	-0.88711600
O	1.25823900	-1.75348900	1.22225100
H	0.18803600	-1.64965800	1.38188200
H	1.44534000	-2.69421800	1.13664500

TS-4 for MPD formation from MDPY:

C	0.16876600	1.16048100	-0.16423100
C	-0.48026200	2.36734300	-0.74277200
H	-1.24993100	2.74968600	-0.07378400
H	0.29311100	3.13909700	-0.84396300
H	-0.90361500	2.17349400	-1.72778600
C	0.62362100	1.04134100	1.26972300
H	1.27003800	1.90332000	1.47119300
H	-0.20834800	1.09515000	1.96950200
C	1.41019700	-0.27770800	1.30738100
H	2.36811400	-0.20656700	1.82181700
H	0.84149000	-1.08715600	1.77100500
C	1.63024900	-0.63246700	-0.14796800
O	2.30413000	-1.49188900	-0.64800500
N	0.86532500	0.30065000	-0.90507500
C	-1.93428200	-0.72863500	-0.00099600
H	-1.30130000	0.27242700	0.01137200
O	-3.08068000	-0.57182700	0.42767800
O	-1.28388200	-1.68182100	-0.43894900
H	0.80688900	0.23816900	-1.91643700

TS-5 for FPAC formation:

C	-0.24364200	-0.32188900	0.52243900
C	-0.00286300	-1.51713900	1.38746800
H	-0.05519300	-2.43320800	0.80117900
H	1.01691000	-1.44795800	1.78294600
H	-0.70038200	-1.56852700	2.22345200
C	0.45401200	-0.21082400	-0.81098700
H	0.60769000	-1.21860400	-1.19426700
H	-0.17627000	0.33618300	-1.50766600
C	1.81042700	0.52659800	-0.74603600

H	2.06738900	0.83719500	-1.76387200
H	1.73919400	1.44685600	-0.16214100
C	2.96714800	-0.31357600	-0.23668600
O	2.91382800	-1.49615700	-0.00335200
O	4.13651600	0.34028300	-0.07131500
H	-1.14655800	0.60208500	2.05701400
N	-0.76890300	0.75120800	1.12604000
C	-1.13421100	2.00192000	0.56424500
H	-1.75438500	2.57511900	1.26252000
O	-0.78143300	2.39365300	-0.51496600
C	-2.92462600	-0.75940900	-0.40331500
H	-1.77886200	-0.91489900	-0.18063600
O	-3.35878600	0.29274500	0.08098600
O	-3.41667000	-1.67914500	-1.06441600
H	4.05767100	1.28219900	-0.27909000

TS-6 for FPAM formation:

C	-1.41052200	1.05330700	-0.63967600
C	-1.88034900	2.01011600	0.45613300
H	-1.37976400	2.97024700	0.31537500
H	-2.95684800	2.18384500	0.39664300
H	-1.64421300	1.65515100	1.46129200
C	0.12402500	0.88121300	-0.68690100
H	0.37219300	0.23762700	-1.53446600
H	0.54334700	1.86995000	-0.90234600
C	0.73029800	0.30326300	0.59545900
H	0.66803300	1.03548000	1.40956800
H	0.17485000	-0.57640500	0.92625300
C	2.18454700	-0.11997800	0.57359300
O	2.77528400	-0.58334200	1.51861900
O	2.15285600	-1.59623400	-0.95841400
H	1.29819600	-1.90091300	-1.28169600
N	-2.06520300	-0.26691500	-0.57737300
C	-2.91991900	-0.74543600	0.34459100
H	-3.13346500	-0.05308600	1.16818200
O	-3.43498700	-1.86353300	0.29732100
H	-1.87033000	-0.89657900	-1.34788100
H	-1.69975300	1.49117500	-1.60047500
N	3.06505200	0.56685100	-0.49730700
H	2.94785200	-0.17883600	-1.24266200
H	4.02462700	0.62961000	-0.15893500
H	2.74263700	1.48313900	-0.80549600

TS-7 for MPD formation from FPAM:

C	1.35166300	-0.63262200	0.32941100
C	2.27135500	-1.56938000	-0.44180500
H	2.45020200	-1.19695800	-1.45448100
H	3.23479600	-1.63805300	0.06870400
H	1.85131300	-2.57576900	-0.50590100
C	1.86032800	0.81720400	0.47639000
H	2.56339400	0.90587500	1.30555900
H	2.37558500	1.12504700	-0.43845800
C	0.58977800	1.65338200	0.67934400
H	0.22822600	1.54789900	1.70495800
H	0.72022700	2.71288100	0.46372600
C	-0.38792000	1.04524700	-0.34328300
O	-0.62881600	1.55704400	-1.44636900
N	0.01174200	-0.48259800	-0.31815800
H	-0.03308800	-0.86781200	-1.26096500
H	1.16763700	-1.04385900	1.32753300
C	-1.38339600	-0.95204900	0.50191100
H	-0.99030700	-1.16963100	1.51018500
O	-2.10335900	-1.77903000	-0.10708000
N	-1.84348600	0.51978800	0.49253900
H	-2.64023700	0.63226000	-0.13194900
H	-2.05898400	0.91298700	1.40620500

IM2-1W:

C	-0.75434100	0.56400800	0.03371500
C	-1.59209900	1.76231300	-0.42157700
H	-1.87359400	2.37712800	0.43680200
H	-1.03050200	2.38384500	-1.12384300
H	-2.50194300	1.41404200	-0.91583400
O	-0.38534300	-0.14212900	-1.16920300
C	0.50697000	1.05088600	0.78099500
H	0.97686200	1.84460000	0.19656900
H	0.19251700	1.49878100	1.72794500
C	1.54369900	-0.04338300	1.06105000
H	2.17084500	0.25698700	1.90845600
H	1.05400700	-0.97433300	1.35671900
C	2.51516800	-0.27883700	-0.08109300
O	2.94404700	0.58269800	-0.81067900
O	2.97276800	-1.54607300	-0.23145600
H	2.55042300	-2.14439000	0.39979700
N	-1.48149600	-0.38712500	0.87096600
H	-1.58652700	-0.03537800	1.81740800
H	-2.41623800	-0.55913500	0.50034600
O	-4.37309300	-1.16084000	-0.10832700

H	-4.96638600	-0.52006100	-0.51693900
H	-4.46794900	-1.95828400	-0.64196400
H	-0.31446300	-1.07310500	-0.92345200

TS for IM2 formation:

C	0.97652900	-0.58425900	-0.18728700
C	1.95018500	-1.32663600	-1.10471200
H	2.15368400	-2.33894600	-0.74684700
H	1.51118200	-1.39196300	-2.10077900
H	2.89328500	-0.78091700	-1.18000600
O	0.72389400	0.71213100	-0.65944500
C	-0.33486600	-1.36630300	-0.01326300
H	-0.69648200	-1.62110000	-1.01305000
H	-0.09619800	-2.31515300	0.47348400
C	-1.46396700	-0.68299900	0.79031100
H	-2.11526000	-1.44957800	1.20836700
H	-1.07012900	-0.12433500	1.64803400
C	-2.37350000	0.27473200	0.02602600
O	-3.58472400	0.23974000	0.13935500
O	-1.79343500	1.17752300	-0.75894600
H	-0.78856500	1.07904100	-0.73854000
N	1.68189200	-0.40548200	1.16312300
H	1.02065700	-0.23285500	1.91928700
H	2.24401000	-1.21570800	1.41786500
O	2.57469000	1.81548700	0.43549000
H	2.30717900	0.54065600	1.01443600
H	3.41057100	1.88831200	-0.03695200
H	1.56313300	1.35465000	-0.32525100

TS for IM3 formation:

C	-1.06170100	-0.92682100	-0.14383100
C	-1.26308900	-2.25609200	0.49573600
H	-2.14133300	-2.77006900	0.10662900
H	-0.38142400	-2.88269600	0.32770900
H	-1.36647500	-2.11906200	1.57243900
O	-2.63685500	0.55357000	1.19884100
C	0.07949600	-0.05404700	0.29313100
H	-0.11394400	0.97464900	-0.00489500
H	0.15181900	-0.08613000	1.37885900
C	1.40419900	-0.53527500	-0.33037600
H	1.62950900	-1.56819400	-0.04730600
H	1.35016600	-0.51581100	-1.42359500
C	2.57535800	0.32875800	0.09947000
O	2.50330200	1.27120400	0.84838400

O	3.77696300	-0.01474000	-0.41628200
H	3.71065600	-0.78376900	-0.99977100
N	-1.71996300	-0.61231000	-1.21543200
H	-2.47130200	-1.21007000	-1.53787400
H	-1.75791500	0.37941800	-1.50178900
O	-2.27171800	2.02701800	-0.75448800
H	-3.09653600	2.42695300	-1.04728800
H	-2.48298700	1.46223400	0.15020200
H	-2.36959900	0.94315800	2.03829200

IM3-1W:

C	1.96972900	0.48018500	-0.01064900
C	3.38571800	0.85291000	-0.34056700
H	4.07418200	0.24599900	0.25534900
H	3.59742800	0.63361800	-1.39109900
H	3.58058800	1.90811600	-0.14695500
C	1.52827400	-0.91350400	-0.40086500
H	1.29940000	-0.89765700	-1.47399900
H	2.38850800	-1.58049500	-0.30125700
C	0.36091300	-1.52982300	0.39752800
H	0.33197600	-2.60060400	0.19999100
H	0.55094200	-1.40135500	1.46877800
C	-1.04111200	-1.00223900	0.11583000
O	-1.96151600	-1.73028600	-0.20553600
O	-1.23264500	0.30597900	0.27870200
H	-0.33371100	0.78816400	0.49213300
N	1.13489200	1.26351400	0.55800900
H	1.51151200	2.18556200	0.77163700
O	-3.66917100	1.71106000	-0.30905700
H	-4.38625800	1.06944700	-0.34581200
H	-2.87329300	1.18540900	-0.11536800

TS for IM3 to MDPY:

C	-1.23258100	0.57877200	-0.15737600
C	-1.85307700	1.92014000	-0.17193000
H	-1.22015400	2.66489400	-0.65076900
H	-2.81213100	1.86153700	-0.69558200
H	-2.07519600	2.22406900	0.85510700
C	-1.83936000	-0.63281200	0.48295600
H	-1.98263400	-0.41108600	1.54442000
H	-2.83023500	-0.82728500	0.06539200
C	-0.82410400	-1.75749000	0.23584400
H	-0.48102900	-2.23459000	1.15027700
H	-1.21618600	-2.52918700	-0.43055200

C	0.33897300	-1.08496500	-0.46841200
O	1.25569600	-1.56665700	-1.07868700
N	-0.07909600	0.31080000	-0.66660900
H	2.00341000	-0.80019400	1.70271300
O	1.35158900	-0.14050100	1.44147900
H	0.63139900	1.01082700	-0.94143600
O	2.14433500	1.72913600	-0.07008000
H	1.95238100	0.96034200	0.61251400
H	3.03197500	1.58739300	-0.41401800

References:

S1. a) A. D. Becke, *J. Chem. Phys.* **1993**, *98*, 5648-5652; b) M. Frisch, G. Trucks, H. Schlegel, G. Scuseria, M. Robb, J. Cheeseman, G. Scalmani, V. Barone, B. Mennucci, G. Petersson, *Inc., Wallingford, CT* **2010**, *6492*; c) M. Cossi, V. Barone, B. Mennucci, J. Tomasi, *Chem. Phys. Lett.* **1998**, *286*, 253-260.

S2. X. L. Du, L. He, S. Zhao, Y. M. Liu, Y. Cao, H. Y. He, K. N. Fan, *Angew. Chem. Int. Ed.* **2011**, *50*, 7815-7819.

S3. Y. B. Huang, J. J. Dai, X. J. Deng, Y. C. Qu, Q. X. Guo, Y. Fu, *ChemSusChem* **2011**, *4*, 1578-1581.

S4. Y. Wei, C. Wang, X. Jiang, D. Xue, J. Li, J. Xiao, *Chem. Commun.* **2013**, *49*, 5408-5410.

S5. A. S. Touchy, S. M. A. Hakim Siddiki, K. Kon, K. I. Shimizu, *ACS Catal.* **2014**, *4*, 3045-3050.

S6. J. D. Vidal, M. J. Climent, P. Concepcion, A. Corma, S. Iborra, M. J. Sabater, *ACS Catal.* **2015**, *5*, 5812-5821.

S7. G. Chieffi, M. Braun, D. Esposito, *ChemSusChem* **2015**, *8*, 3590-3594.

S8. A. Ledoux, L. S. Kuigwa, E. Framery, B. Andrioletti, *Green Chem.* **2015**, *17*, 3251-3254.

S9. C. Ortiz-Cervantes, M. Flores-Alamo, J. J. García, *Tetrahedron Lett.* **2016**, *57*, 766-771.

S10. G. Gao, P. Sun, Y. Li, F. Wang, Z. Zhao, Y. Qin, F. Li, *ACS Catal.* **2017**, *7*, 4927-4935.

S11. C. Wu, H. Zhang, B. Yu, Y. Chen, Z. Ke, S. Guo, Z. Liu, *ACS Catal.* **2017**, *7*, 7772-7776.

S12. J. J. Martínez, L. Silva, H. A. Rojas, G. P. Romanelli, L. A. Santos, T. C. Ramalho, M. H. Brijaldod, F. B. Passos, *Catal. Today* **2017**, *296*, 118-126.

S13. S. M. A. H. Siddiki, A. S. Touchy, A. Bhosale, T. Toyao, Y. Mahara, J. Ohyama, A. Satsuma, K. I. Shimizu, *ChemCatChem* **2018**, *10*, 789-795.

S14. Z. Xu, P. Yan, H. Jiang, K. Liu, Z. C. Zhang, *Chin. J. Chem.* **2017**, *35*, 581-585.

S15. S. Wang, H. Huang, C. Bruneau, C. Fischmeister, *ChemSusChem* **2017**, *10*, 4150-4154.

S16. C. Wu, X. Luo, H. Zhang, X. Liu, G. Ji, Z. Liu, Z. Liu, *Green Chem.* **2017**, *19*, 3525-3529.

S17. A. S. Amarasekara, Y. M. Lawrence, *Tetrahedron Lett.* **2018**, *59*, 1832-1835.

S18. P. Cao, T. Ma, H. Y. Zhang, G. Yin, J. Zhao, Y. Zhang, *Catal. Commun.* **2018**, *116*, 85-90.

S19. G. Metzker, R. M. Dias, A. C. Burtoloso, *ChemistrySelect* **2018**, *3*, 368-372.

S20. Y. Wei, C. Wang, X. Jiang, D. Xue, Z. T. Liu, J. Xiao, *Green Chem.* **2014**, *16*, 1093-1096.

S21. G. Metzker, R. M. P. Dias, A. C. B. Burtoloso, *ChemistrySelect* **2018**, *3*, 368-372.

S22. C. Xie, J. Song, H. Wu, Y. Hu, H. Liu, Z. Zhang, P. Zhang, B. Chen, B. Han, *J. Am. Chem. Soc.* **2019**, *141*, 4002-4009.

S23. S. Huh, S. Y. Hong, S. Chang, *Org. Lett.* **2019**, *21*, 2808-2812.

S24. W. Y. Yu, Q. Xing, C. M. Chan, Y. W. Yeung, *J. Am. Chem. Soc.* **2019**, *141*, 3849-3853.

S25. Y. Wei, C. Wang, X. Jiang, D. Xue, Z. T. Liu, J. Xiao, *Green Chem.* **2014**, *16*, 1093-1096.

S26. Á. Mourelle-Insua, L. A. Zampieri, I. Lavandera, V. Gotor-Fernández, *Adv. Syn. Catal.* **2018**, *360*, 686-695.

S27. C. Zhang, Y. Ding, Y. Gao, S. Li, G. Li, *Org. Lett.* **2018**, *20*, 2595-2598.
